# Two New Indole Derivatives from the Beibu Gulf Coral-Derived Fungus *Pestalotiopsis microspora* GXIMD 02530

**DOI:** 10.3390/md24080279

**Published:** 2026-08-12

**Authors:** Hui Shi, Guihua Yang, Xiaolin Liu, Miaoping Lin, Humu Lu, Chenghai Gao, Yonghong Liu, Xiaowei Luo

**Affiliations:** Guangxi Key Laboratory of Marine Drugs, University Engineering Research Center of High-Efficient Utilization of Marine Traditional Chinese Medicine Resources, Guangxi, Technology Innovation Center for the Development and Utilization of Marine Drugs and Bioproducts for Beibu Gulf, Ministry of Natural Resources, Institute of Marine Drugs, Guangxi University of Chinese Medicine, Nanning 530200, China

**Keywords:** marine fungus, *Pestalotiopsis microspora*, indole derivatives, bioactivity

## Abstract

Two new indole derivatives (**1** and **2**), along with fourteen known indole and polyketide compounds, were characterized from the Beibu Gulf coral-derived fungus *Pestalotiopsis microspora* GXIMD 02530. Their structures and absolute configurations were determined by comprehensive spectroscopic analysis, electronic circular dichroism (ECD) calculations, and single-crystal X-ray diffraction. Structurally, furoindolin A (**1**) was obtained as a rare racemic 6/5/5 tricyclic indole derivative incorporating a dihydrofuranone ring, which was further separated into a pair of enantiomers by chiral chromatographic resolution. Compounds (±)-**1**, **3**, **4**, and **6**–**12** exhibited inhibition of LPS-induced NF-κB luciferase activities. Phomopsilactone (**10**) displayed antibacterial activities against *Staphylococcus epidermidis*, *Bacillus subtilis*, and *Staphylococcus aureus*, with MIC values of 15.6, 31.25, and 62.5 μg/mL, respectively. Our findings would expand the chemical variety of indole derivatives and highlight furoindolin A (+)-**1** as a promising chemical template for further biosynthetic and anti-inflammatory pharmacological investigation.

## 1. Introduction

Natural products have always been a significant source for drug discovery owing to their diverse chemical structures and biological activities [1,2,3]. Marine microorganisms, especially marine fungi, have been evidenced as predominant sources of structurally diverse compounds with significant pharmacological activity in recent years [4]. The Beibu Gulf in the northern part of the South China Sea remains largely underexplored for marine microorganisms and organisms inhabiting four representative marine ecosystems, which have been attracting increasing attention in marine natural product discovery [5]. A total of 477 new marine natural products were obtained from the Beibu Gulf before September 2022, including polyketides, terpenoids, nitrogen-containing compounds, and glucosides, which were found with cytotoxic, antibacterial, and anti-inflammatory activities [5]. The *Pestalotiopsis* species widely reside in terrestrial and oceanic habitats and are known as potential producers of structurally unique and biologically active secondary metabolites, which have continued to attract great interest from both natural product chemists and pharmacologists [6,7].

Indole is known as a prestigious natural scaffold structure made up of a benzene ring and a pyrrole ring, which is widely found in both naturally occurring and biologically active compounds with wide sources, including plants, animals, marine microorganisms and organisms [8,9]. The FDA has approved over 40 indole-containing agents for the treatment of various diseases in recent decades, and the development of indole-related drugs has attracted considerable attention from medicinal chemists [10]. Notably, an unprecedented oxidized di-*seco*-indole diterpenoid, peniditerpenoid A, was recently found as an osteoclast differentiation inhibitor from a mangrove-sediment-derived *Penicillium* sp. by our group [11]. Additionally, a rare class of indole-diterpenoid derivatives with uncommon 3-methyl-3-hydroxybutyl substituents, brefeldindoles A−F, was obtained from the Beibu Gulf mangrove-derived fungus *Penicillium brefeldianum* GXIMD 02511 in our recent study, which could also suppress RANKL-induced osteoclast differentiation without observed cytotoxicity in bone marrow macrophages, suggesting anti-osteoporosis potential lead compounds [12].

**Figure 1:**
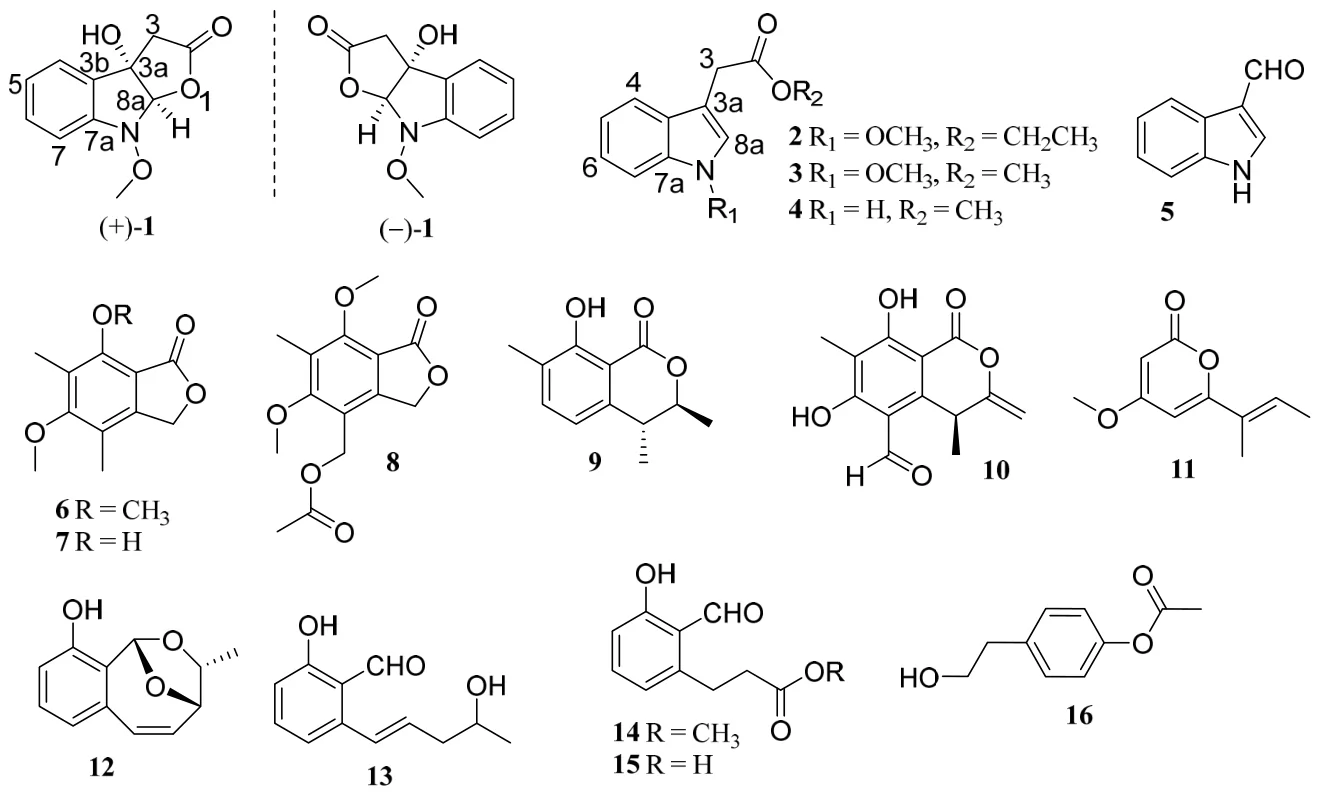
Chemical structures of compounds **1**–**16**.

In our ongoing studies to search for biologically active compounds from the Beibu Gulf-derived marine fungi, a series of structurally new and bioactive compounds have been obtained, including anti-osteoclastogenic indole alkaloids [11,12], azaphilones [13], chlorinated phenolic derivatives [14,15], antiproliferative ascochlorins [16,17], and resorcylic acid lactones [18]. The Beibu Gulf coral-derived fungus *Pestalotiopsis microspora* GXIMD 02530 attracted our attention based on its interesting HPLC-UV profiles of the extract. In this study, two new indole derivatives and fourteen known compounds were identified from *P. microspora* GXIMD 02530 (Figure 1). Details of the isolation, structural elucidation, and bioactive profiles of these compounds are reported herein.

## 2. Results

### 2.1. Structure Elucidation

Compound **1** was isolated as a white powder with the molecular formula C_11_H_11_NO_4_, as determined by the HR-ESIMS ion peak at 222.0760 [M + H]^+^ (calculated for C_11_H_12_NO_4_^+^ 222.0761), indicating seven degrees of unsaturation (DOUs). The UV spectrum of **1** demonstrated typical UV absorptions of indole chromophores at approximately 235 and 284 nm. The ^1^H NMR data (Table 1) displayed signals, including a hydroxyl group [*δ*_H_ 6.67 (1H, br s, 3a-OH)], one methoxy group [*δ*_H_ 3.88 (3H, s, *N*-OCH_3_)], one methylene [*δ*_H_ 2.85 (2H, s, H_2_-3)], and one highly deshielded methine [*δ*_H_ 5.76 (1H, s, H-8a)], along with four aromatic methines [*δ*_H_ 7.34 (1H, d, *J* = 7.5 Hz, H-4), 7.10 (1H, t, *J* = 7.5 Hz, H-5), 7.35 (1H, t, *J* = 7.5 Hz, H-6), 7.05 (1H, d, *J* = 7.5 Hz, H-6)], indicative of a 1,2-substituted benzene. Apart from the above-mentioned proton-containing groups, there were four remaining quaternary carbons in the ^13^C NMR data of **1**, including one carbonyl (*δ*_C_ 175.2), two aromatics (*δ*_C_ 147.2, 130.0), and one oxygenated (*δ*_C_ 79.9). The above spectral data of **1** revealed an indole derivative, the structure of which resembled the epoxidation product of indole 3-acetic acid [19].

**Figure 2:**
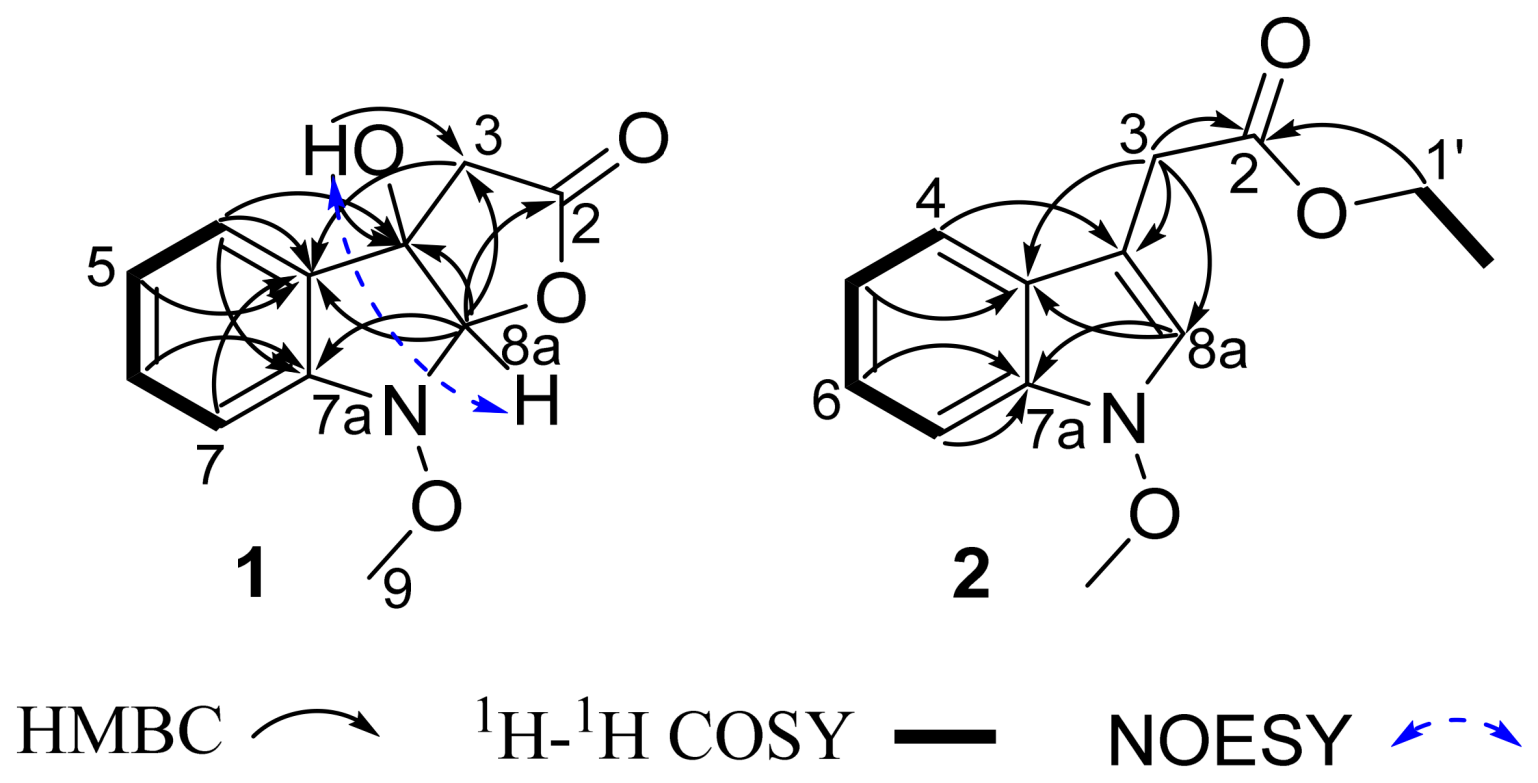
Key HMBC, ^1^H–^1^H COSY and/or NOESY correlations of **1** and **2**.

The sequential ^1^H–^1^H COSY correlations of H-4/H-5/H-6/H-7 and the HMBC correlations (Figure 2) of H-4/C-3a, 3b, 7a; H-7/C-3b, C-7a; and H-8a/C-3a, C-3b, C-7a allowed the establishment of an oxygenated indoline unit. The above-mentioned structural units or functional groups accounted for six DOUs; the remaining one DOU therefore required an additional ring in the molecule. The HMBC correlations of H-8a/C-2, C-3, C-3a and 3a-OH/C-3 permitted the existence of a dihydrofuranone ring fused to the indoline moiety. Meanwhile, the methoxy group (*δ*_H/C_ 3.89/63.6) was deduced to be attached at the *N* atom, as in the same case of co-isolated methyl 1-methoxyindole-3-acetate (**3**). Thus, compound **1** was identified as an unusual 6/5/5 tricyclic indole derivative incorporating a rare dihydrofuranone ring and was given the trivial name furoindolin A. Moreover, the HR-ESI-MS ion peak at 244.0583 [M + Na]^+^ (calculated for C_11_H_11_NO_4_Na^+^, 244.0586) could be found in the HPLC-MS data of the crude extracts of *P. microspora* GXIMD 02530, which suggests that furoindolin A (**1**) is probably a natural product.

**Figure 3:**
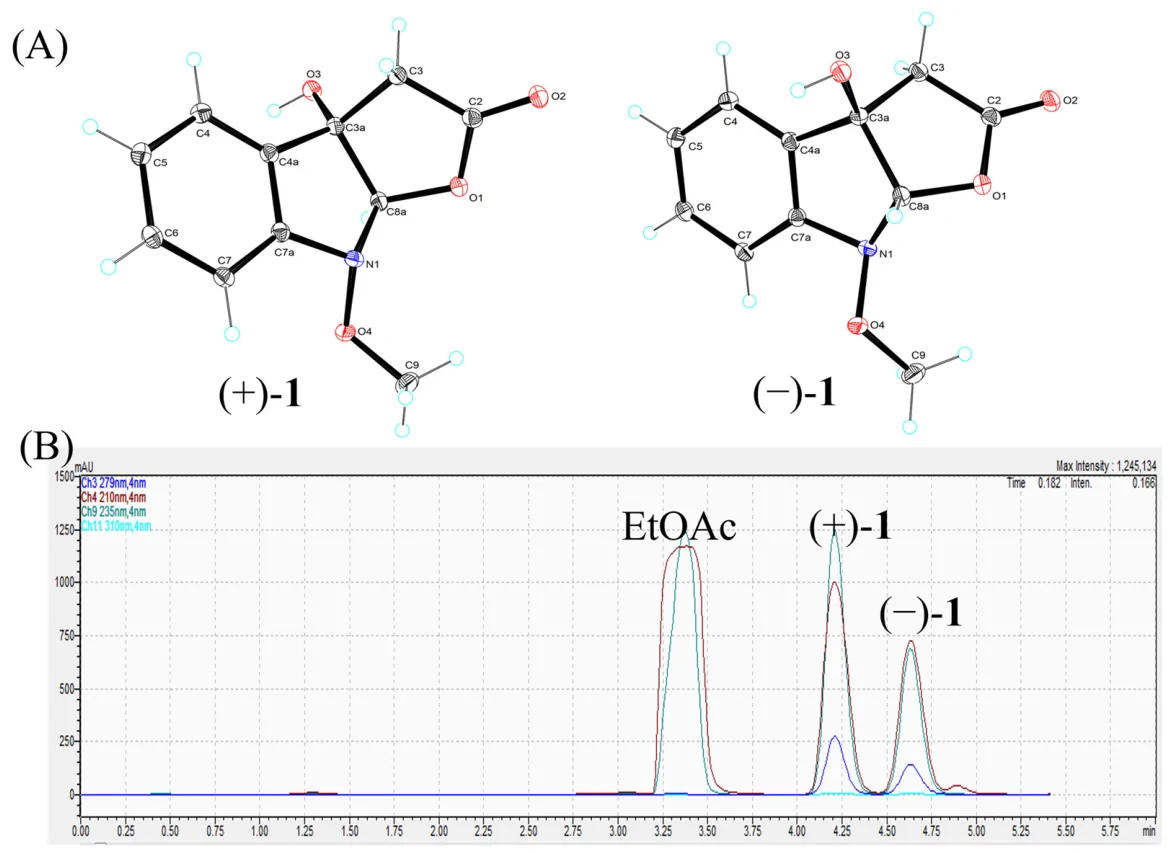
X-ray crystallographic structures (**A**) and chiral HPLC resolution (**B**) of (±)-**1**.

**Figure 4:**
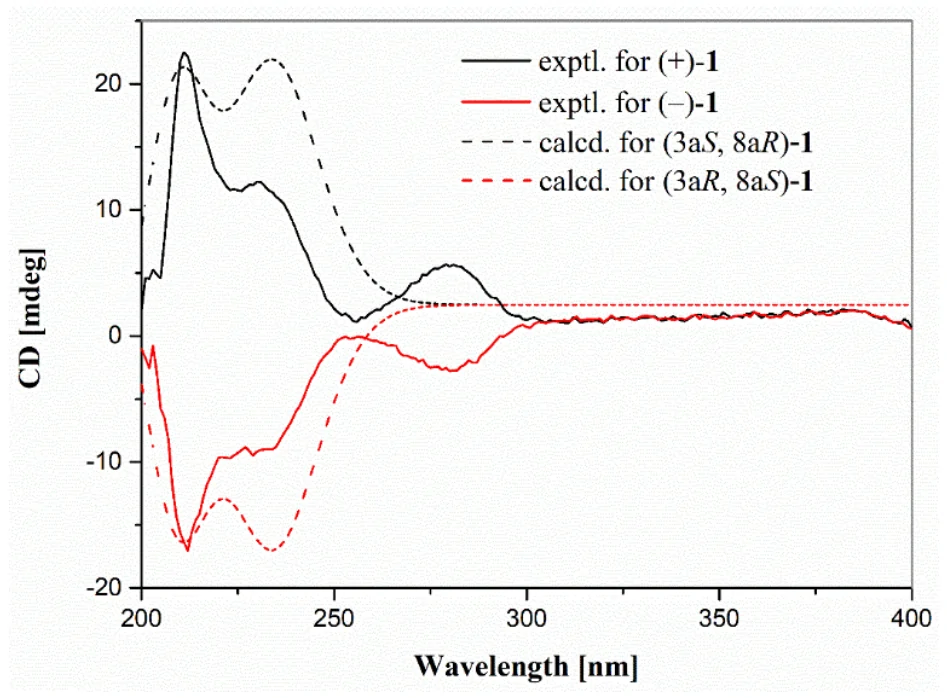
Experimental and calculated ECD spectra of (±)-**1**.

The NOESY correlation (Figure 2) of 3a-OH/H-8a suggested both are located on the same face. Interestingly, single crystals of **1** were obtained from slow evaporation in methanol, which unequivocally confirmed the above-assigned planar structure as well as the relative configuration of **1**. However, the centrosymmetric space group (*P*2_1_/c) of **1** (Figure 3A), along with the barely measurable optical rotation value and marginal CD effects, explicitly revealed a racemic mixture. Thus, compound **1** was further separated by chromatographic resolution using chiral HPLC columns (Figure 3B). The absolute configurations of (±)-**1** were further determined by comparing the calculated ECD curves with the experimental ECD ones (Figure 4). The enantiomers (+)-**1** and (−)-**1** were further determined with (3a*S*, 8a*R*) and (3a*R*, 8a*S*) configurations, respectively.

Compound **2** was obtained as a colorless oil with the molecular formula C_13_H_15_NO_3_ as supported by the HR-ESIMS data (*m*/*z* 234.1123 [M + H]^+^ (calcd for C_13_H_16_NO_3_^+^, 234.1125)). The 1D NMR data (Table 1), in conjunction with the UV spectrum of **2**, also revealed an indole derivative, whose structure closely resembled that of co-isolated methyl 1-methoxyindole-3-acetate (**3**). The key difference was the presence of an ethyl group (*δ*_H/C_ 4.16/61.0, CH_2_-1′; 1.25/14.4, CH_3_-2′) bonded at the carbonyl group in **2** instead of a methyl group in **3**, which was confirmed by the ^1^H–^1^H COSY correlations of H_2_-1′/H_3_-2′ and HMBC correlation (Figure 2) of H-1′/C-2. Based on the above discussion, the structure of **2** was established and was designated ethyl 2-(1-methoxy-1*H*-indol-3-yl) acetate.

The other known compounds were mainly identified by comparison of spectroscopic data with literature data, which were methyl 1-methoxyindole-3-acetate (**3**) [20], methyl indole-3-acetate (**4**) [20], 1*H*-indole-3-carboxaldehyde (**5**) [21], 5,7-dimethoxy-4,6-dime-thylphthalide (**6**) [22], 7-hydroxy-5-methoxy-4,6-dimethylisobenzofuran-1(3*H*)-one (**7**) [22], pestalotiophthalide B (**8**) [22], gamahorin (**9**) [23], phomopsilactone (**10**) [24], pestalopyrone (**11**) [25], cladoacetal A (**12**) [26], clavisinol E (**13**) [27], methyl-(2-formyl-3-hydroxyphenyl) propanoate (**14**) [28], 3-(2-formyl-3-hydroxyphenyl)-propionic acid (**15**) [26], and 2-*p*-acetoxyphenylethanol (**16**) [29], respectively.

### 2.2. Biological Activities

**Figure 5:**
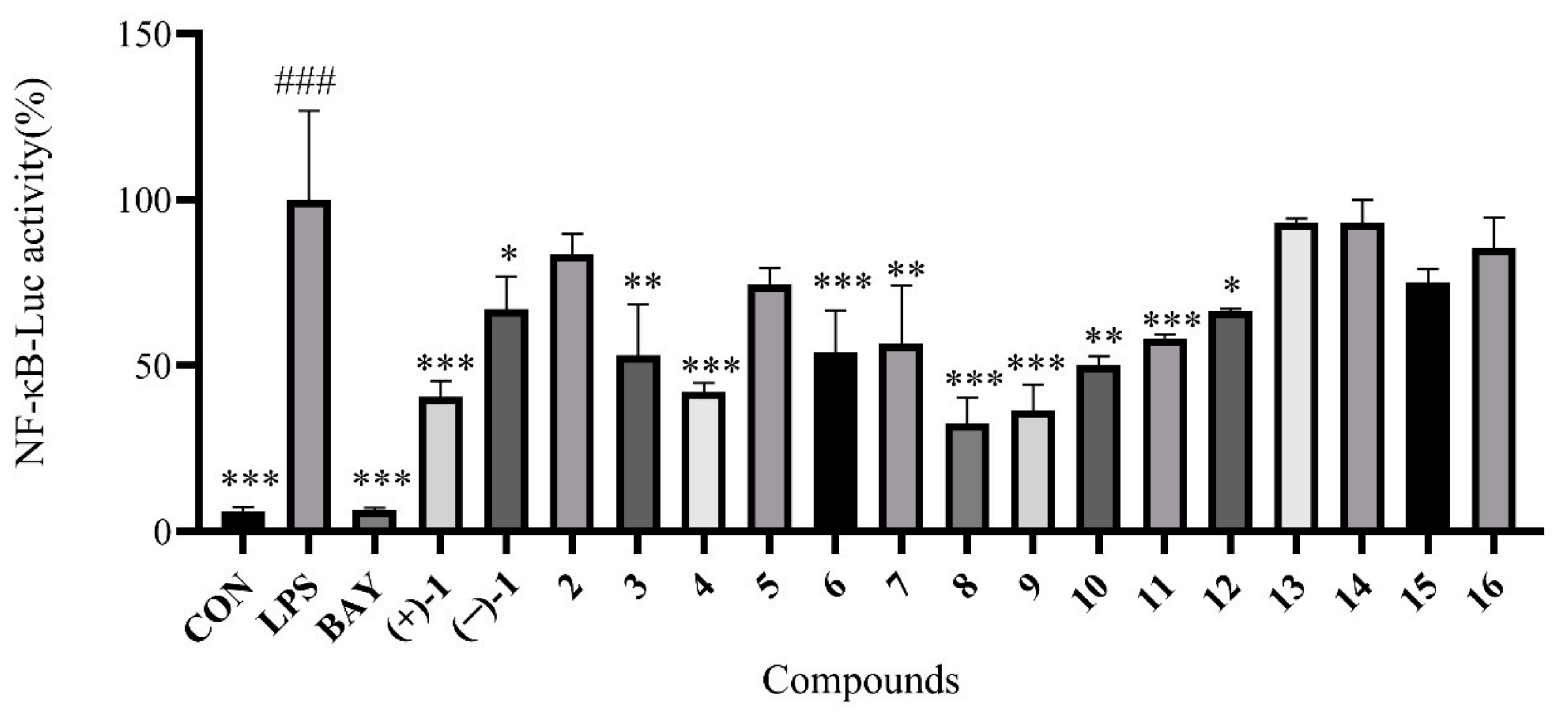
The NF-κB activation inhibitory effects of compounds **1**–**16** in RAW264.7 cells at 20 μM. *n* = 3. The luciferase activities were measured. ### *p* < 0.001 relative to untreated controls, * *p* < 0.05, ** *p* < 0.01, and *** *p* < 0.001 compared to LPS-treated controls. BAY (BAY11-7082 treated, positive control).

During our ongoing search for anti-osteoclastogenic compounds from marine fungi [12,13], the obtained compounds were primarily tested for their inhibitory activities of lipopolysaccharide (LPS)-induced NF-κB activation in RAW264.7 macrophages at 20 μM by NF-κB luciferase reporter gene. Among them, compounds (±)-**1**, **3**, **4**, and **6**–**12** displayed inhibition of LPS-induced NF-κB luciferase activities in RAW 264.7 macrophage cells (Figure 5). Their structure−activity relationships were preliminarily discussed herein. Interestingly, compound (+)-**1** showed greater NF-κB luciferase inhibitory activities than that of (−)-**1**, suggesting that the chiral centers play a pivotal role in the activity. The absent activity of **2** compared with those of **3** and **4** revealed that a methoxy group at C-2 is helpful for the effect.

**Figure 6:**
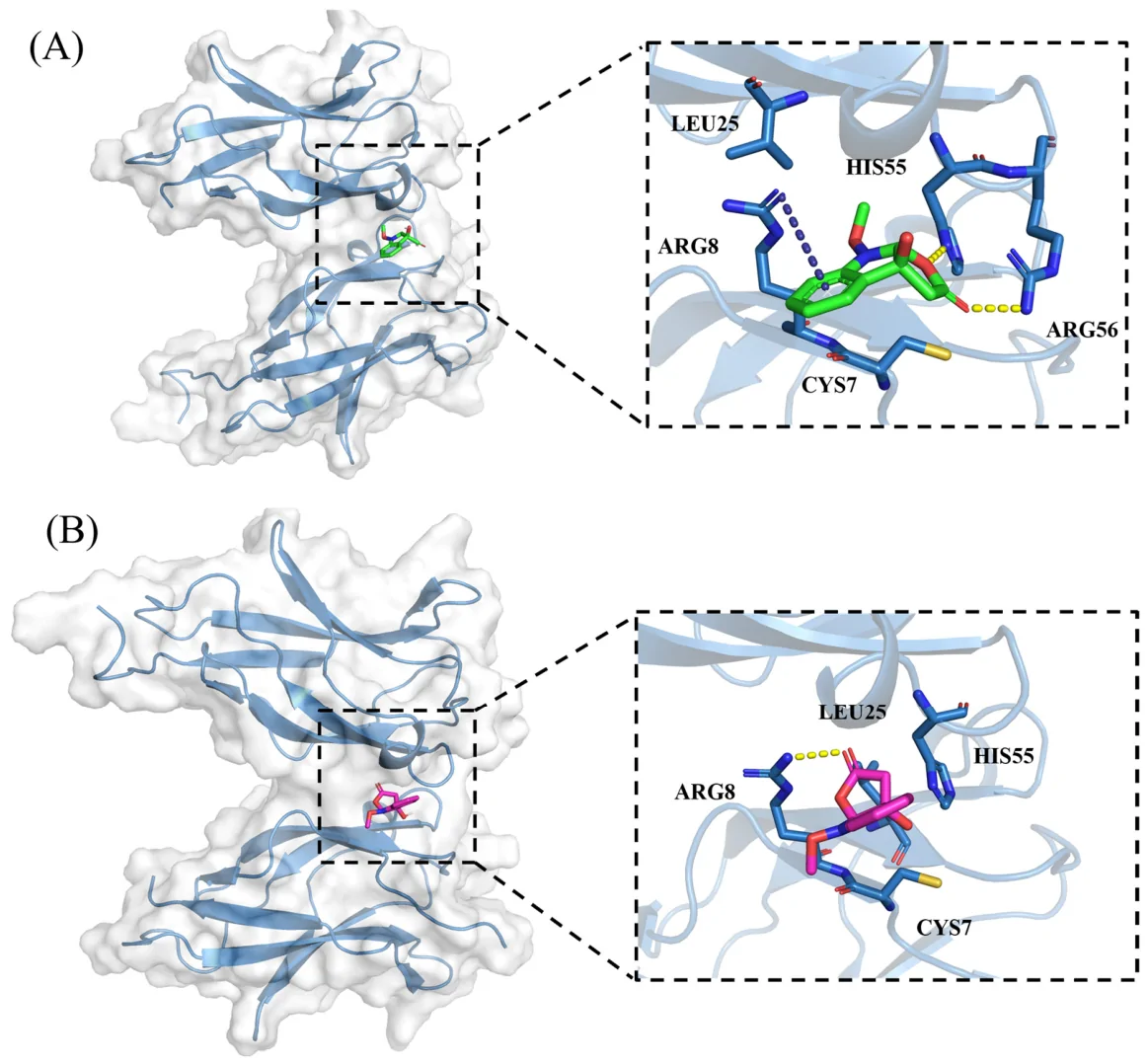
The predicted binding modes of compound (+)-**1** (**A**) and (−)-**1** (**B**) with the NF-κB p65 protein (PDB code: 1MY5) by molecular docking, as shown by the cartoon and the highlighted interacting residues are shown by thick sticks. The yellow dashed lines represent hydrogen bonds. The blue dashed line represents p-π interaction.

The molecular docking study was performed to further investigate the binding modes of potential (+)-**1** and (−)-**1** with NF-κB p65. The theoretical binding modes of (+)-**1** and (−)-**1** with the NF-κB p65 protein (PDB code: 1MY5) were shown in Figure 6 with the binding scores of −5.3 and −4.9 kcal/mol, respectively, which agreed with the aforementioned NF-κB luciferase activity of (+)-**1** and (−)-**1**. Detailed analysis showed that the dihydrofuranone unit in (+)-**1** tightly interacts via hydrogen bonds with the surrounding amino acid residues HIS55 and ARG56. Additionally, the benzene ring in (+)-**1** could interact with ARG8 by π-π stacking interaction. However, the carbonyl group in (−)-**1** just forms a hydrogen bond with residue ARG8. Both (+)-**1** and (−)-**1** could interact with LEU25 and CYS7 via hydrophobic interactions.

Additionally, compounds **1**–**16** were also evaluated for antibacterial activities by the broth dilution method. Amongst them, phomopsilactone (**10**) displayed antibacterial activities against *Staphylococcus epidermidis*, *Bacillus subtilis*, and *Staphylococcus aureus*, with MIC values of 15.6, 31.25, and 62.5 μg/mL, respectively. However, none of them showed cytotoxicity against five human cancer cell lines at 20 μM, including DLD-1, HeLa, HT-29, NCI-H460, and HepG2 cells. None of them showed significant DPPH radical scavenging activity (100 μg/mL) and α-glucosidase inhibitory activities (250 μg/mL).

## 3. Materials and Methods

### 3.1. General Experimental Procedures

Optical rotation was collected on an IP-digi300/3 polarimeter (Shanghai InsMark Instrument Technology Co., Ltd., Shanghai, China). ECD spectra were performed on a JASCO J-1500 spectropolarimeter (JASCO Corporation, Tokyo, Japan). NMR spectra were measured on a Bruker Avance spectrometer (Bruker BioSpin, Fällanden, Switzerland) operating at 500 MHz for ^1^H and 125 MHz for ^13^C, with tetramethylsilane (TMS) as the internal standard. HR-ESIMS data were collected using a SCIEX Triple TOF mass spectrometer (SCIEX, Framingham, MA, USA). The absorbance was recorded by a Victor Nivo multimode plate reader (PerkinElmer, Waltham, MA, USA). TLC was performed on precoated silica gel GF_254_ plates (10–40 µm) and column chromatography (CC) on silica gel (200–300 mesh) (Qingdao Marine Chemical Factory, Qingdao, China). Semi-preparative HPLC was performed on a Shimadzu SCL-10VAP system (Shimadzu, Tokyo, Japan) equipped with a YMC ODS-A column (10 × 250 mm, 5 µm) at a flow rate of 2 mL/min. Artificial sea salt was purchased from Guangzhou Haili Aquarium Technology Co., Ltd. (Guangzhou, China) All solvents were of analytical grade and purchased from Shanghai Titan Scientific Co., Ltd. (Shanghai, China).

### 3.2. Fungal Material

The fungal strain GXIMD 02530 was isolated from the coral *Porites lutea* collected from the Weizhou Islands, Guangxi Zhuang Autonomous Region, China, and was cultured on potato dextrose agar (PDA) at 28 °C for 5 days. It was identified as *Pestalotiopsis microspora* GXIMD 02530 (original number: BBG14) based on the BLAST (Basic Local Alignment Search Tool) analysis of internal spacer (ITS) sequences (GenBank accession no. ON460248) in the NCBI database as previously described [30]. The voucher specimen has been deposited at the Institute of Marine Drugs, Guangxi University of Chinese Medicine.

### 3.3. Fermentation, Extraction, and Isolation

The strain GXIMD 02530 was first incubated in malt extract broth (MB) medium (15 g of malt extract powder, 20 g of artificial sea salt, and 1 L of tap-distilled water, at pH 7.4–7.8) for 3 days. Its mycelium was then transferred to malt extract broth (MB) medium (200 mL) and was cultured on a rotary shaker at 180 r/min for 3 days to obtain the seed cultures. Large-scale fermentation was carried out on solid rice medium in 80 Erlenmeyer flasks (1 L, each containing 120 g of rice, 3 g of artificial sea salt, 3 g of corn steep liquor, 1.5 g of methionine, and 150 mL of water, at pH 7.4–7.8). The flasks were autoclaved at 121 °C for 25 min before inoculation, and each was inoculated with 10 mL of spore suspension and statically cultured at room temperature for 30 days. The cultures were finally extracted three times with ethyl acetate to yield crude extracts (664 g).

The crude extracts were initially fractionated by medium-pressure liquid chromatography (MPLC) on a petroleum ether–CH_2_Cl_2_ gradient system (1:0–0:1, *v*/*v*), followed by elution with CH_2_Cl_2_–CH_3_OH (1:0–1:1, *v*/*v*), to afford nine fractions (Fr.1–Fr.9). Fr.2 was chromatographed on an ODS column with stepwise gradient elution using CH_3_OH–H_2_O (10~100%), yielding twelve subfractions (Fr.2.1–Fr.2.12). Fr.2.6 was separated by semi-preparative HPLC eluted with 70% CH_3_CN–H_2_O at 2 mL/min, giving compounds **2** (7.8 mg, *t*_R_ 24 min) and **9** (21.0 mg, *t*_R_ 25 min). Fr.2.7 was also purified by semi-preparative HPLC eluted with 70% CH_3_CN–H_2_O at 2 mL/min to afford compound **10** (27.6 mg, *t*_R_ 26 min). Fr.3 was subjected to an ODS column by stepwise gradient elution with CH_3_OH–H_2_O (10~100%), producing twelve subfractions (Fr.3.1–Fr.3.12). Fr.3.7 was purified by semi-preparative HPLC eluted with 40% CH_3_CN–H_2_O at 2 mL/min, yielding compounds **7** (46 mg, *t*_R_ 19 min) and **4** (4.8 mg, *t*_R_ 19.5 min). Fr.3.10 was isolated by semi-preparative HPLC using 50% CH_3_CN–H_2_O to provide compound **3** (7.6 mg, *t*_R_ 22 min).

Fr.5 was fractionated on an ODS column by stepwise gradient elution with CH_3_OH–H_2_O (10~100%), affording 27 subfractions (Fr.5.1–Fr.5.27). Fr.5.6 was further divided by an ODS column with stepwise gradient elution of CH_3_OH–H_2_O (10~100%) to give 20 subfractions (Fr.5.6.1–Fr.5.6.20). Compound **13** (13.9 mg, *t*_R_ 18.5 min) was obtained from Fr.5.6.14 by semi-preparative HPLC eluted with 50% CH_3_OH–H_2_O at 2 mL/min. Fr.5.6.10 was purified by semi-preparative HPLC (50% CH_3_OH–H_2_O, 2 mL/min) to afford compounds **12** (14 mg, *t*_R_ 17.5 min) and **14** (3 mg, *t*_R_ 20 min), along with four subfractions. Fr.5.6.10.2 was further re-purified on an ODS column with 30% CH_3_CN–H_2_O at 2 mL/min to yield compound **5** (14.7 mg, *t*_R_ 15 min). Fr.5.6.10.3 was subjected to a phenyl column (40% CH_3_CN–H_2_O, 2 mL/min) to give compound **15** (45 mg, *t*_R_ 12.5 min). Compounds **1** (20.3 mg, *t*_R_ 16 min) and **16** (86 mg, *t*_R_ 16 min) were obtained from Fr.5.6.10.5 by an ODS column eluted with 45% CH_3_CN–H_2_O at 2 mL/min. Fr.5.14 was further separated by semi-preparative HPLC to give three subfractions. Fr.5.14.3 was purified on an ODS column (65% CH_3_CN–H_2_O, 2 mL/min) to yield compound **11** (19 mg, *t*_R_ 19 min). Fr.5.14.4 was also purified by an ODS column (65% CH_3_CN–H_2_O, 2 mL/min) to give compound **8** (12 mg, *t*_R_ 19.7 min). Fr.5.14.5 was purified on an ODS column with 70% CH_3_OH–H_2_O at 2 mL/min, affording compound **6** (22 mg, *t*_R_ 20 min).

Compound **1** was obtained as a racemic mixture and was further resolved on a chiral Phenomenex column (Lux cellulose-1, 4.6 × 250 mm, 5 µm) (Phenomenex Inc., Torrance, CA, USA) eluted with 40% isopropanol/n-hexane at 1 mL/min, giving a pair of enantiomers, (+)-**1** (4.6 mg, *t*_R_ 4.0 min) and (−)-**1** (2.5 mg, *t*_R_ 4.5 min).

Furoindolin A (+)-**1**: white powder; [α${]}_{D}^{25}$+102 (*c* 0.2, CH_3_OH); UV (CH_3_OH) λmax (log ε) 203 (3.84), 235 (3.23), 284 (2.53) nm; ECD (0.25 mg/mL, CH_3_OH) λmax(∆ε) 211 (+22.5), 231 (+12.2), 255 (+1.15), 281 (+5.64) nm; (−)-**1**: [α${]}_{D}^{25}$−100 (*c* 0.2, CH_3_OH); ECD (0.25 mg/mL, CH_3_OH) λmax(∆ε) 212 (−17.1), 229 (−9.48), 256 (−0.06), 280 (−2.77) nm; ^1^H NMR and ^13^C NMR data, see Table 1; HR-ESIMS *m*/*z* 222.0760 [M + H]^+^ (calculated for C_11_H_12_NO_4_^+^ 222.0761).

Ethyl 2-(1-methoxy-1*H*-indol-3-yl) acetate (**2**): colorless oil; UV (CH_3_OH) λmax (log ε) 222 (3.51), 282 (2.77) nm; ^1^H and ^13^C NMR data, see Table 1; HR-ESIMS *m*/*z* of 234.1123 [M + H]^+^ (calcd for C_13_H_16_NO_3_^+^, 234.1125).

### 3.4. ECD Calculations

The theoretically calculated ECD spectra of (±)-**1** were performed by the Gaussian 16 software (Gaussian Inc., Wallingford, CT, USA) according to our previously reported method [13,16]. Four conformers were generated by Molecular Merck force field (MMFF) calculations via Spartan’14 software (Wavefunction Inc., Irvine, CA, USA), which were further subjected to time-dependent DFT calculations at the M062X/def2TZVP//B3LYP/6-31+G (d) level in methanol by adopting 50 excited states. ECD spectra were generated using the program SpecDis 3.0 (University of Wurzburg, Wurzburg, Germany) by applying Gaussian band shapes of 0.3 eV and were shifted by −16 nm to facilitate comparison to the experimental data.

### 3.5. X-Ray Crystallography

The crystallographic data of compound (±)-**1** were obtained by the solvent diffusion method, which was collected on a Rigaku XtaLAB PRO single-crystal diffractometer (Rigaku Corporation, Tokyo, Japan) using Cu Ka radiation (λ = 1.54178 Å). Briefly, the X-ray crystal structure was solved using SHELXS97, expanded by difference Fourier techniques, and finally refined by full-matrix least-squares calculation. The non-hydrogen atoms were refined anisotropically, and all hydrogen atoms were fixed at the geometrically ideal positions.

Crystal data for furoindolin A (±)-**1**: C_11_H_11_NO_4_, *M*r = 221.21, crystal size 0.08 × 0.07 × 0.05 mm^3^, monoclinic, a = 8.2829(2) Å, b = 16.1218(3) Å, c = 8.1864(2) Å, *α* = 90°, *β* = 113.670(3)°, *γ* = 90°, *V* = 1001.21(4) Å^3^, *T* = 99.9(8) K, space group *P*21/c, Z = 4, *μ*(CuK*α*) = 0.953 mm^−1^, 4969 reflections collected, 1963 independent reflections (*R*_int_ = 0.0308). The final *R*_1_ values were 0.0353 (*I* > 2σ(*I*)). The final w*R*(*F*^2^) values were 0.0857 (*I* > 2σ(*I*)). The final *R*_1_ values were 0.0434 (all data). The final w*R*(*F*^2^) values were 0.0893 (all data). The goodness of fit on F^2^ was 1.042. The crystallographic data for the structure of (±)-**1** have been deposited in the Cambridge Crystallographic Data Centre (deposition number: CCDC 2571697).

### 3.6. Biological Assays

#### 3.6.1. NF-κB Luciferase Assay

Compounds **1**–**16** were evaluated for NF-κB luciferase activity by luciferase reporter gene assay as previously described [12,13]. Briefly, RAW264.7 cells, stably transfected with an NF-κB luciferase reporter construct, were treated with compounds **1**–**16** (20 μM) and a typical NF-κB inhibitor BAY11-7082 (5 μM, positive control) (Sigma-Aldrich, Darmstadt, Germany) for 4 h, followed by stimulation with LPS (100 ng/mL, Sigma-Aldrich) for 6 h. The luciferase activity was determined by the luciferase assay system (Promega, Madison, WI, USA).

#### 3.6.2. Antibacterial Assay

Compounds **1**–**16** were evaluated for antibacterial activities against *Staphylococcus aureus*, *Bacillus subtilis*, and *Staphylococcus epidermidis*, using the disk diffusion method as previously described [30]. Sterile filter paper disks (6 mm in diameter) were impregnated with the compounds (25 μg) and were placed on the inoculated agar surfaces. The plates were then incubated at 37 °C for 18–24 h, after which the diameters of inhibition zones were measured.

The bioactive compound **10** was further subjected to the minimum inhibitory concentration (MIC) test using the broth microdilution method. Serial twofold dilutions of compound **10** were prepared in sterile broth medium in 96-well microtiter plates. Bacterial suspensions were added to each well to achieve a final inoculum of approximately 5 × 10^5^ CFU/mL. The plates were incubated at 37 °C for 18–24 h. Ampicillin functioned as the positive control and showed antibacterial activities against *S. aureus*, *B. subtilis*, and *S. epidermidis* with MIC values of 0.024, 0.024, and 0.050 μg/mL, respectively.

#### 3.6.3. Cytotoxicity Assay

Compounds **1**–**16** were also tested for cytotoxicity against five human cancer cell lines, DLD-1, HeLa, HT-29, NCI-H460, and HepG2 cells (Chinese Academy of Sciences Cell Bank, Shanghai, China) based on our previously reported protocols [16]. In brief, the cells were seeded in 96-well plates and incubated at 37 °C for 12 h, then treated with compounds (20 μM). After 72 h of incubation, cell viability was assessed, and the inhibition rate for each cell line was calculated relative to the untreated control.

#### 3.6.4. Antioxidant Activity Assay

Compounds **1**–**16** were also evaluated for antioxidant effects by the DPPH (1,1-diphenyl-2-picrylhydrazyl) (Beijing Solarbio Science & Technology Co., Ltd., Beijing, China) radical scavenging assay as previously reported [31]. Briefly, the compounds (1000, 500, 100, 50, and 10 μg/mL), along with the positive control ascorbic acid, were mixed with fresh DPPH in ethanol solutions. The absorbance was recorded at 517 nm using a multimode plate reader. The DPPH scavenging rate was calculated based on the absorbance values.

#### 3.6.5. α-Glucosidase Inhibitory Assay

Compounds **1**–**16** were also evaluated for antidiabetic activity by α-glucosidase (Sigma-Aldrich) inhibitory activity using a multimode plate reader as previously reported [32]. Acarbose (Shanghai Titan Scientific Co., Ltd., Shanghai, China) was used as a positive control with an IC_50_ value of 373.3 μmol/L.

#### 3.6.6. Molecular Docking

The crystal structure of NF-κB p65 (PDB ID: 1MY5) was obtained from the Protein Data Bank and used for molecular docking with **1** using AutoDock Vina 1.5.7 [32]. Protein and ligand preparation were performed using AutoDockTools 1.2.0. Polar hydrogen atoms were added to the protein structure, and Kollman charges were assigned to the receptor, whereas Gasteiger charges were assigned to the ligand. The prepared structures were converted into PDBQT format prior to docking. Molecular docking calculations were carried out using AutoDock Vina 1.5.7, which employs a stochastic global optimization algorithm combined with local optimization to explore ligand-binding conformations. The docking search space was defined to encompass the predicted binding pocket of ARF1, and multiple binding poses were generated for each docking simulation. The binding pose with the lowest predicted binding free energy was selected as the representative complex for subsequent analysis. The resulting protein–ligand complex was visualized using PyMOL 2.5.0 software (DeLano Scientific, Palo Alto, CA, USA) to generate 3D interaction figures.

## 4. Conclusions

Two new indole derivatives (**1** and **2**), along with fourteen known indole and polyketide compounds, were characterized from the Beibu Gulf coral-derived fungus *Pestalotiopsis microspore* GXIMD 02530. Their structures were determined by extensive spectroscopic analysis and by comparison with literature data. Notably, compound **1** was obtained as a rare 6/5/5 tricyclic indole derivative incorporating a dihydrofuranone ring, as unequivocally confirmed by X-ray single-crystal X-ray diffraction analysis. Interestingly, compound **1** was defined as a racemic mixture, which was further separated into a pair of enantiomers by chiral chromatographic resolution, absolute configurations of which were assigned by ECD calculations. Among them, compounds (±)-**1**, **3**, **4**, and **6**–**12** exhibited inhibition of LPS-induced NF-κB luciferase activities. Interestingly, compound (+)-**1** showed greater NF-κB luciferase inhibitory activities than that of (−)-**1**, suggesting that the chiral centers play a pivotal role in the activity. Additionally, phomopsilactone (**10**) displayed antibacterial activities against *Staphylococcus epidermidis*, *Bacillus subtilis*, and *Staphylococcus aureus*, with MIC values of 15.6, 31.25, and 62.5 μg/mL, respectively. Our findings would expand the chemical variety of indole derivatives and highlight furoindolin A (+)-**1** as a promising chemical template for further biosynthetic and anti-inflammatory pharmacological investigation.

## Figures and Tables

**Table 1:** ^1^H (500 MHz) and ^13^C (125 MHz) NMR data of **1** (DMSO-*d*_6_) and **2** (CDCl_3_).

Pos.	1		2	
	*δ*_C_, Type	*δ*_H_ (*J* in Hz)	*δ*_C_, Type	*δ*_H_ (*J* in Hz)
2	175.2, C		171.9, C	
3	41.3, CH_2_	2.85, s	31.4, CH_2_	3.71, s
3a	79.9, C		104.5, CH_2_	
3b	130.0, C		123.8, C	
4	123.5, CH	7.34, d (7.5)	119.3, CH	7.58, d (7.5)
5	123.8, CH	7.10, t (7.5)	120.0, CH	7.11, t (7.5)
6	129.9, CH	7.35, t (7.5)	122.7, CH	7.23, t (7.5)
7	113.4, CH	7.05, d (7.5)	108.4, CH	7.41, d (7.5)
7a	147.2, C		132.4, C	
8a	109.6, CH	5.76, s	122.2, CH	7.26, s
9	63.6, CH_3_	3.89, s	65.9, CH_3_	4.05, s
1′			61.0, CH_2_	4.16, q (7.0)
2′			14.4, CH_3_	1.25, t (7.0)
3a-OH		6.67, brs		

## Data Availability

The original data presented in this study are included in the article/Supplementary Materials. Further inquiries can be directed to the corresponding author.

## Supplementary Materials

The following supporting information can be downloaded at: https://www.mdpi.com/article/10.3390/md24080279/s1. The 1D and 2D NMR, HR-ESIMS, and UV spectra of **1** and **2**; ECD calculated data of **1**; physicochemical data of known compounds **3**–**16**; and the ITS sequence of *Pestalotiopsis microspora* GXIMD 02530 have been prepared as electronic attachments.
