# Supplementary material for: Two New Indole Derivatives from the Beibu Gulf Coral-Derived Fungus Pestalotiopsis microspora GXIMD 02530
Source: Mar Drugs. 2026 Aug 12;24(8):279. doi: 10.3390/md24080279 (PMC13514921; doi:10.3390/md24080279)
Supplement: Supplementary file 1 [file marinedrugs-24-00279-s001.zip › marinedrugs-4464764-supplementary.pdf]

# Two new indole derivatives from the Beibu Gulf coral-derived fungus *Pestalotiopsis microspora* GXIMD 02530

Hui Shi †, Guihua Yang †, Xiaolin Liu, Miaoping Lin, Humu Lu, Chenghai Gao, Yonghong Liu, Xiaowei Luo*

Guangxi Key Laboratory of Marine Drugs; University Engineering Research Center of High-Efficient Utilization of Marine Traditional Chinese Medicine Resources, Guangxi; Technology Innovation Center for the Development and Utilization of Marine Drugs and Bioproducts for Beibu Gulf, MNR; Institute of Marine Drugs, Guangxi University of Chinese Medicine, Nanning 530200, China.

Correspondence: luoxiaowei1991@126.com

† These authors contributed equally to this work.

## Contents

**Figure S1**. $^{1}$H NMR spectrum of furoindolin A (**1**) (DMSO-$d_6$, 500 MHz) ......3
**Figure S2.** $^{13}$C NMR spectrum of furoindolin A (**1**) (DMSO-$d_6$, 125 MHz) ......4
**Figure S3**. HSQC spectrum of furoindolin A (**1**) (DMSO-$d_6$) ......5
**Figure S4.** HMBC spectrum of furoindolin A (**1**) (DMSO-$d_6$) ......6
**Figure S5.** $^{1}$H-$^{1}$H COSY spectrum of furoindolin A (**1**) (DMSO-$d_6$). ......7
**Figure S6.** NOESY spectrum of furoindolin A (**1**) (DMSO-$d_6$). ......8
**Figure S7.** $^{1}$H-NMR spectrum of ethyl 2-(1-methoxy-1H-indol-3-yl) acetate (**2**) ($CDCl_3$, 500 MHz) ......9
**Figure S8.** $^{13}$C NMR spectrum of ethyl 2-(1-methoxy-1H-indol-3-yl) acetate (**2**) ($CDCl_3$, 125 MHz) ......10
**Figure S9.** HSQC spectrum of ethyl 2-(1-methoxy-1H-indol-3-yl) acetate (**2**) ($CDCl_3$) ......11
**Figure S10.** HMBC spectrum of ethyl 2-(1-methoxy-1H-indol-3-yl) acetate (**2**) ($CDCl_3$) ......12
**Figure S11.** $^{1}$H-$^{1}$H COSY spectrum of ethyl 2-(1-methoxy-1H-indol-3-yl) acetate (**2**) ($CDCl_3$) ... 13
**Figure S12.** HR-ESIMS spectrum of furoindolin A (**1**) ......14
**Figure S13.** HR-ESIMS spectrum of ethyl 2-(1-methoxy-1*H*-indol-3-yl) acetate (**2**) ......14
**Figure S14.** UV spectrum of furoindolin A (**1**) ......15
**Figure S15.** UV spectrum of ethyl 2-(1-methoxy-1*H*-indol-3-yl) acetate (**2**) ......15
**Table S1**. Energies of **1** at MMFF94 force field ......16
**Table S2**. Energies of **1** at B3LYP/6–31+g(d) level in methanol. ......16
**Figure S16.** The optimized conformers and equilibrium populations of **1**. ......18
**Figure S17**. The HR-ESI-MS ion peak of furoindolin A (**1**) from LC-MS data of the crude extracts of the strain *P. microspora* GXIMD 02530. ......18
**Physicochemical data of known compounds 3–16.** ......18
**Figure S18.** Inhibition zone diameter range (mm) of compounds **9-10** and positive controls against

*B. subtilis*, *S. epidermidis*, and *S. aureus* after 24 h of incubation at 37 °C. ........................................ 22
**The ITS sequence of *Pestalotiopsis microspora* GXIMD 02530** ........................................................ 22

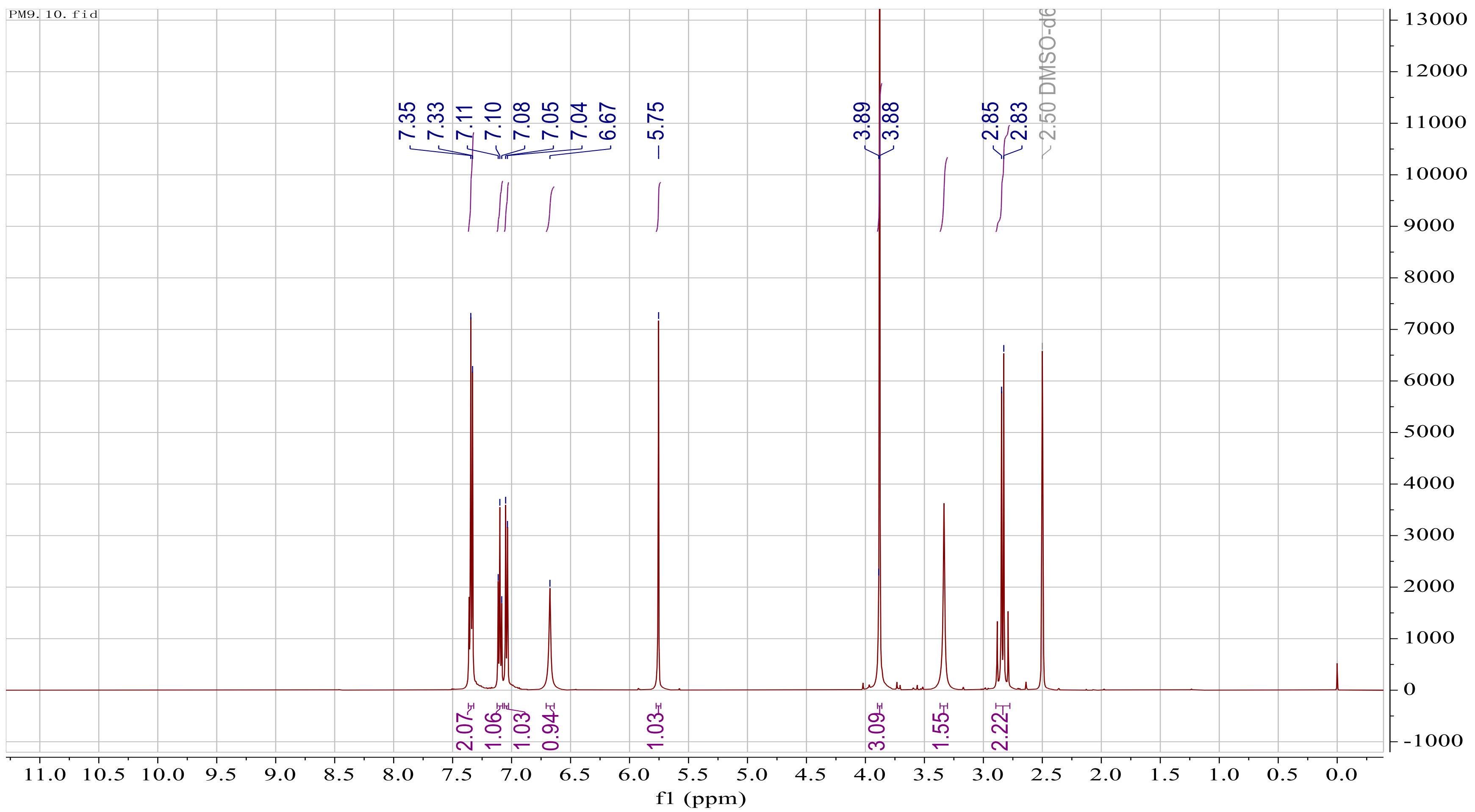


**Figure S1**. $^{1}$H NMR spectrum of furoindolin A (**1**) (DMSO-$d_6$, 500 MHz)

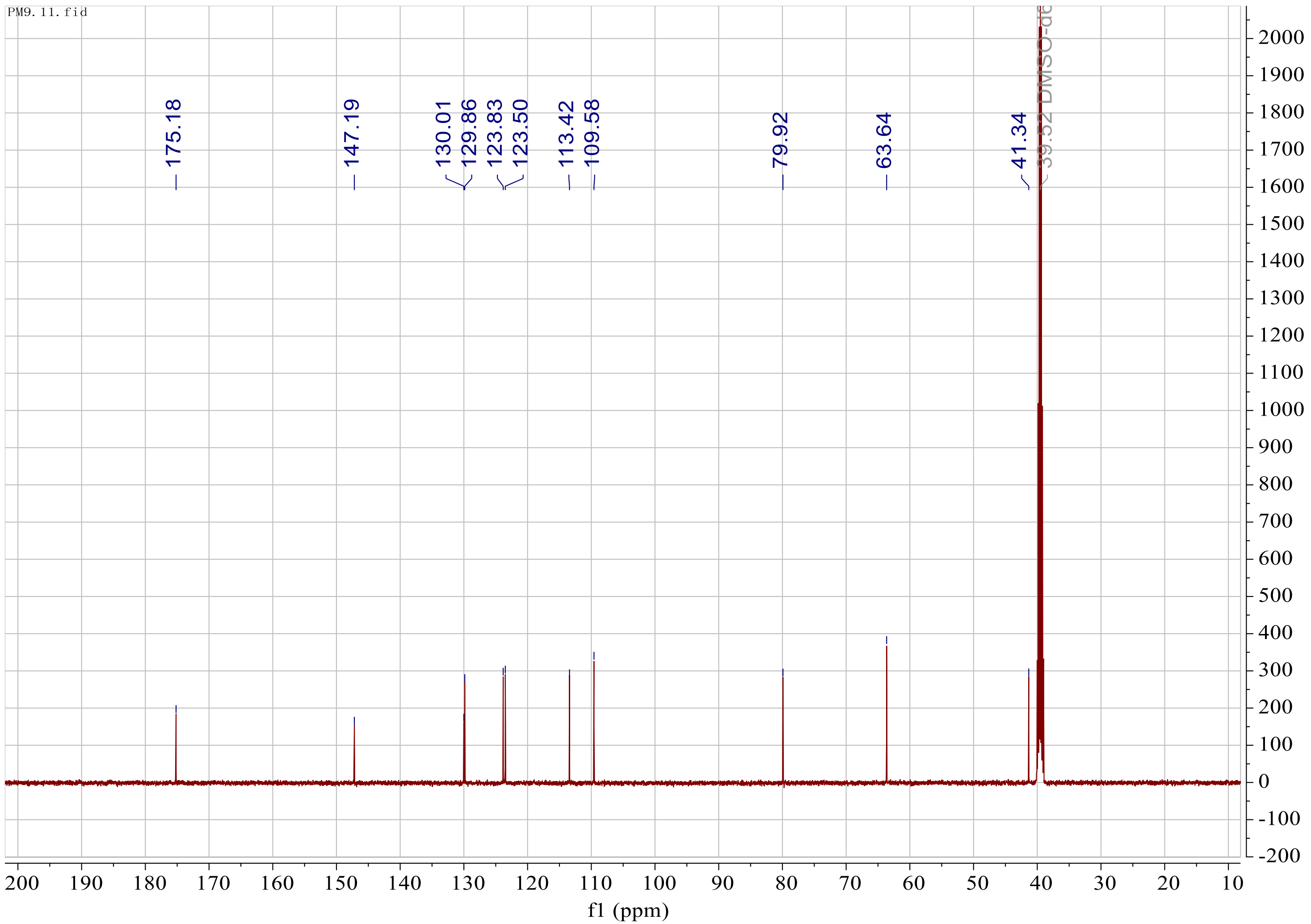


**Figure S2.** $^{13}$C NMR spectrum of furoindolin A (**1**) (DMSO-$d_6$, 125 MHz)

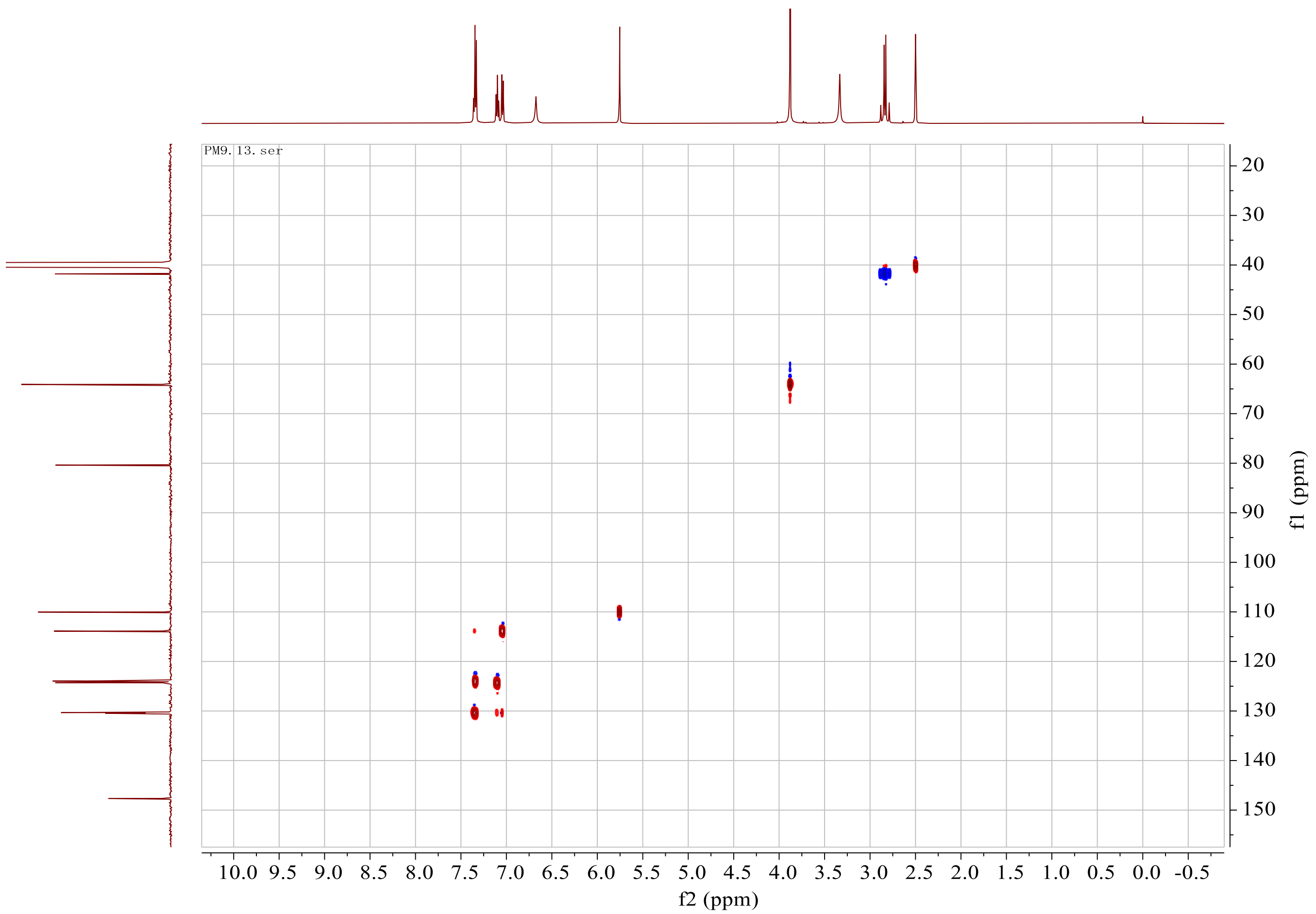


**Figure S3**. HSQC spectrum of furoindolin A (**1**) (DMSO-$d_6$)

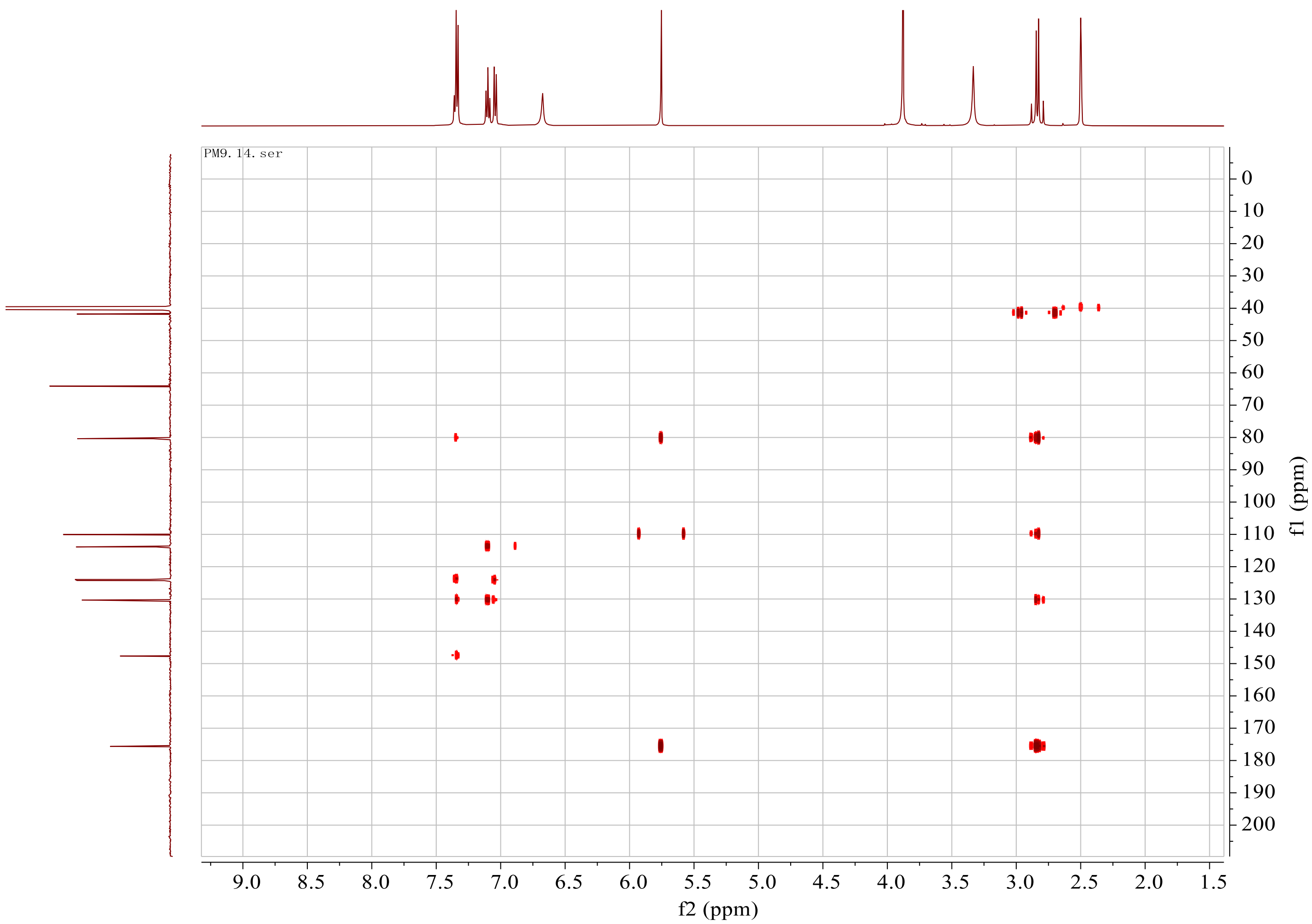


**Figure S4.** HMBC spectrum of furoindolin A (**1**) (DMSO-$d_6$)

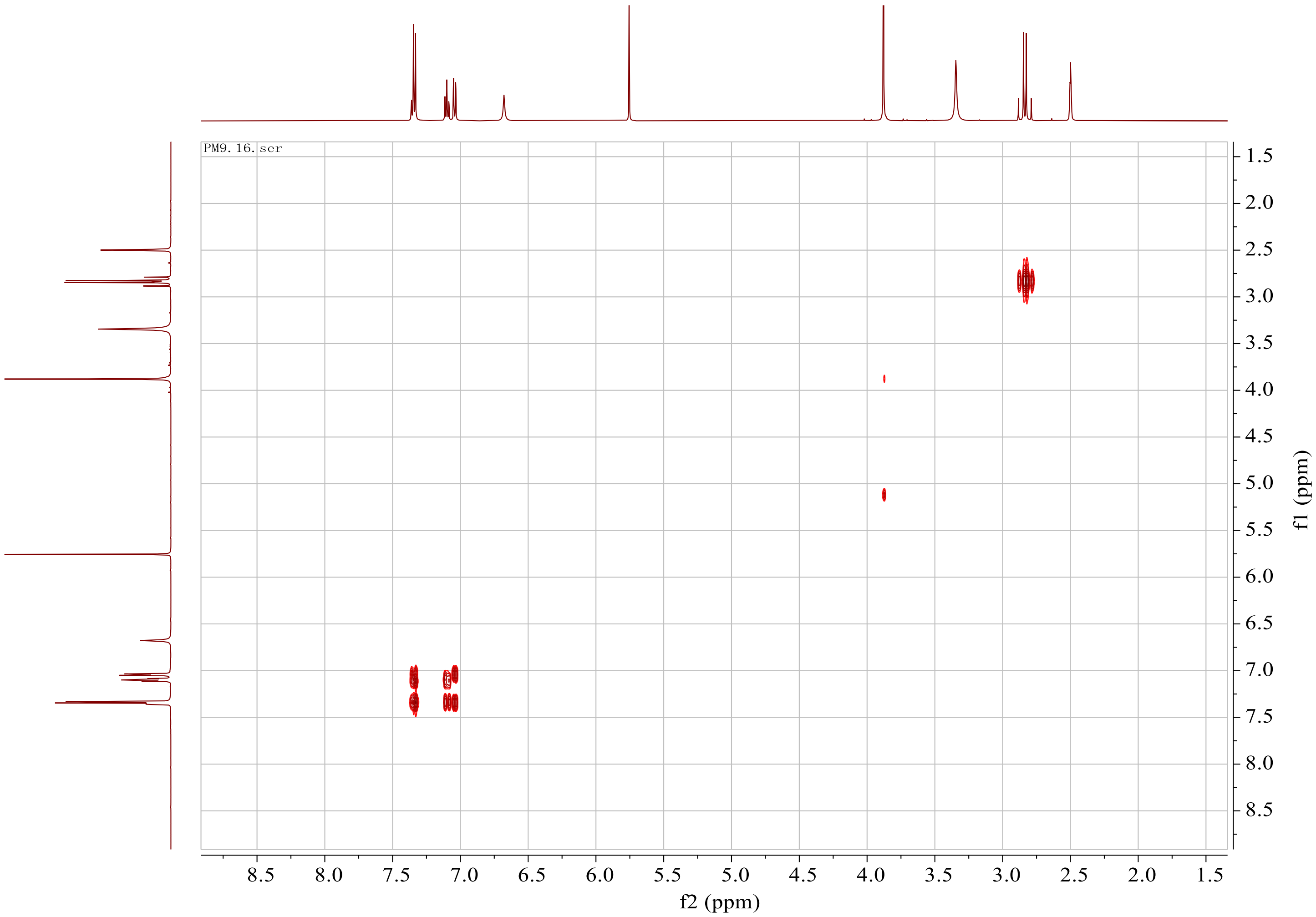


**Figure S5.** $^{1}H$-$^{1}H$ COSY spectrum of furoindolin A (**1**) (DMSO-$d_6$).

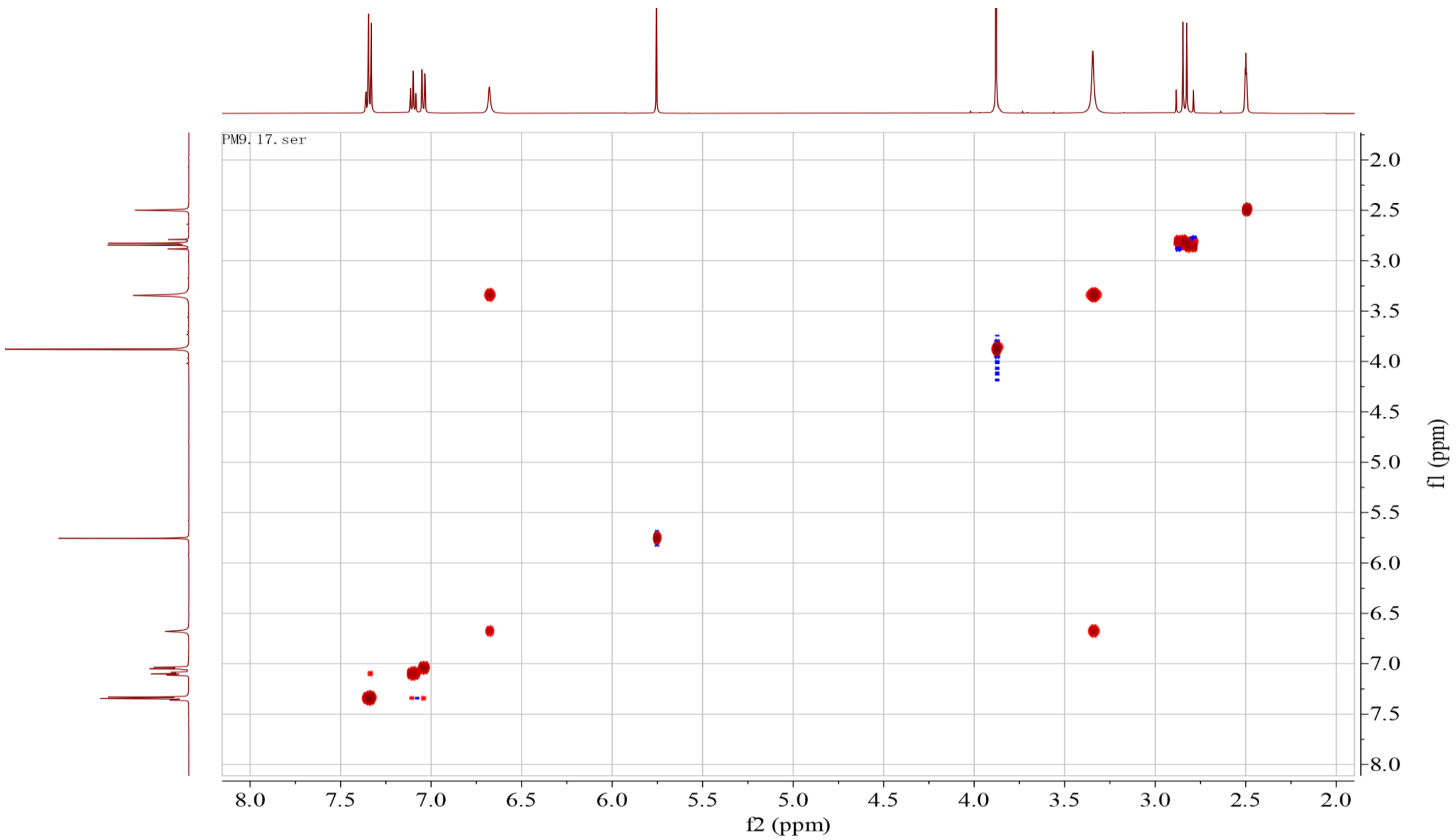


**Figure S6.** NOESY spectrum of furoindolin A (**1**) (DMSO-$d_6$).

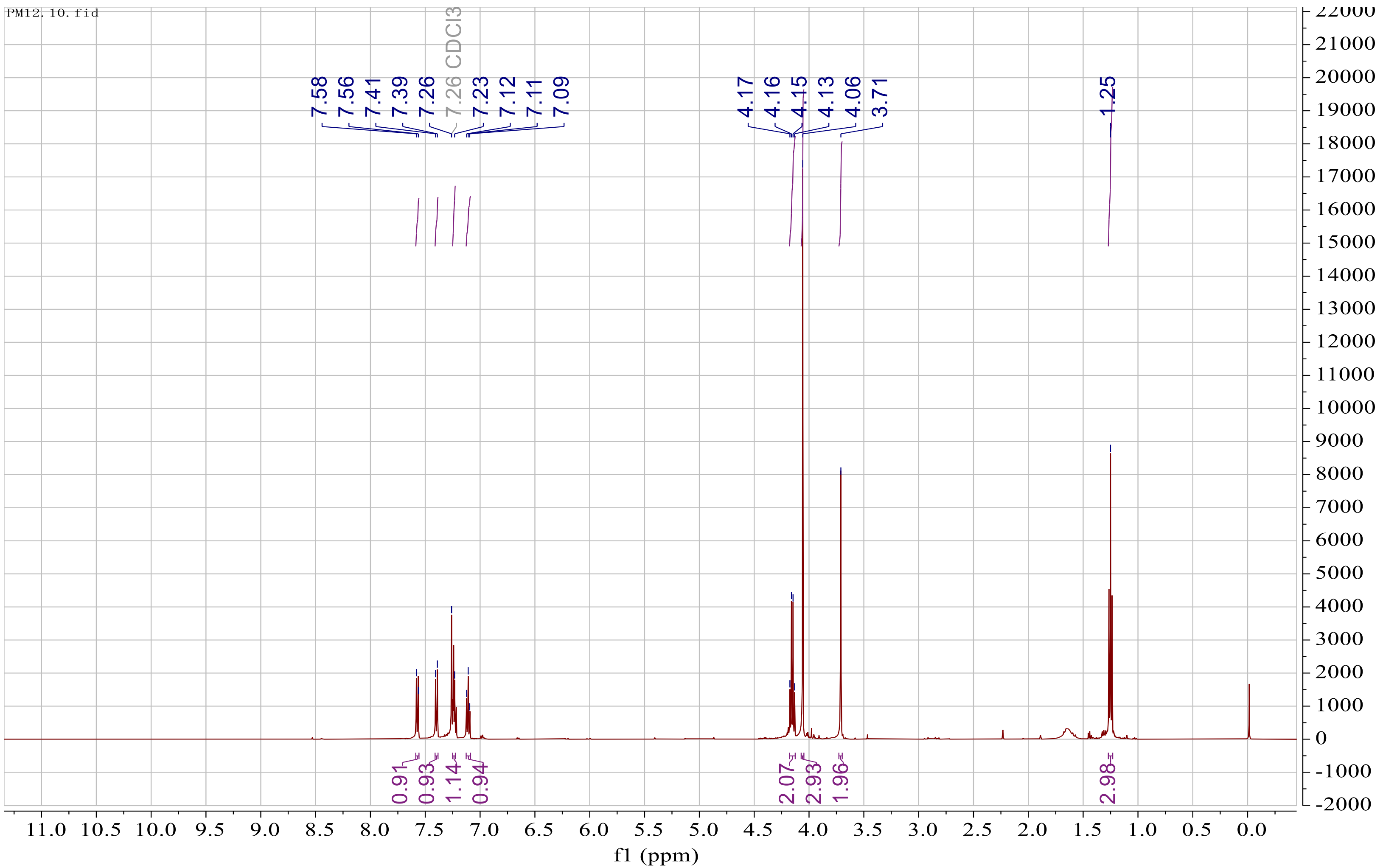


**Figure S7.** $^{1}$H-NMR spectrum of ethyl 2-(1-methoxy-1H-indol-3-yl) acetate (**2**) ($CDCl_3$, 500 MHz)

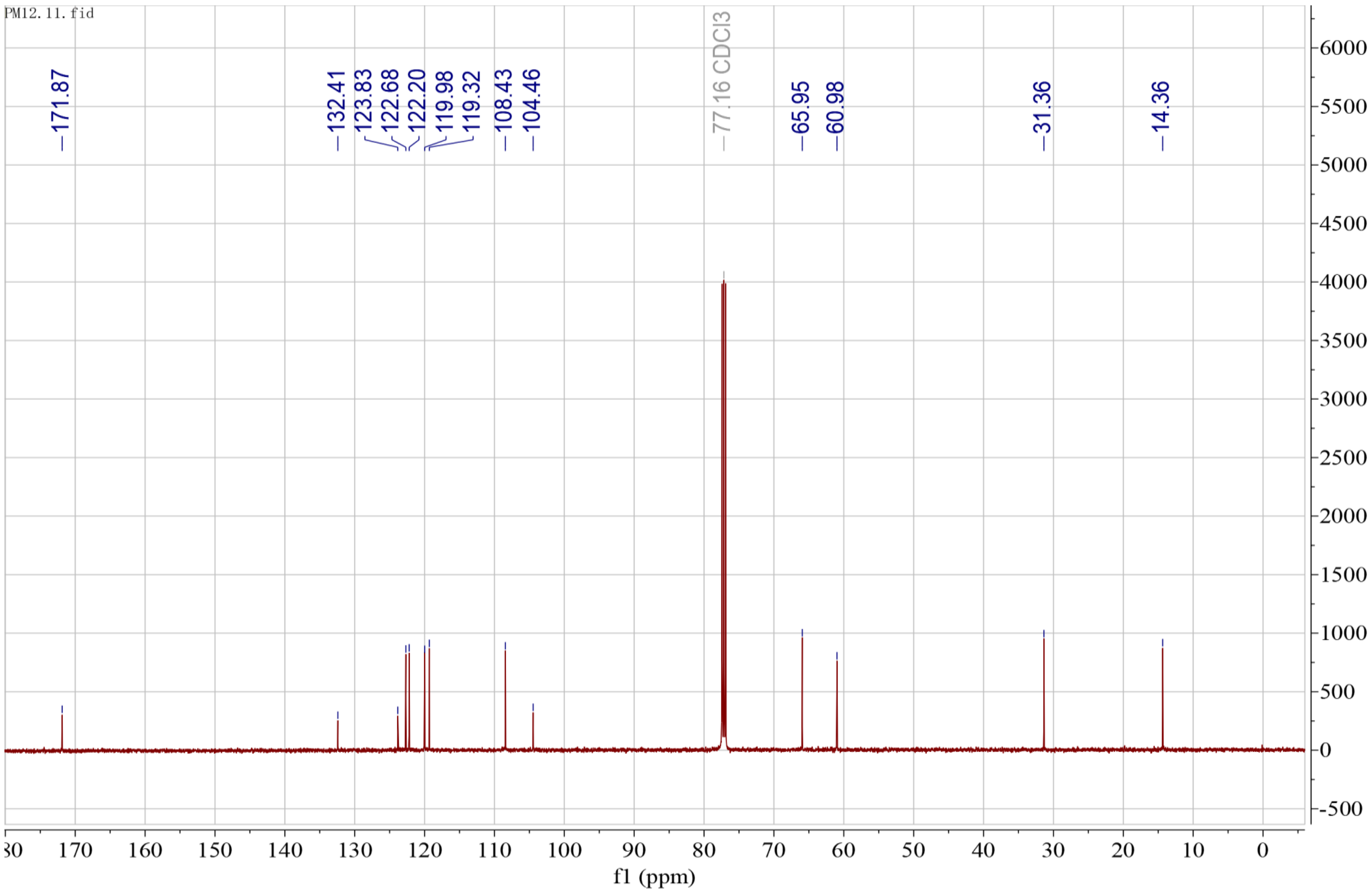


**Figure S8.** $^{13}$C NMR spectrum of ethyl 2-(1-methoxy-1H-indol-3-yl) acetate (**2**) ($CDCl_3$, 125 MHz)

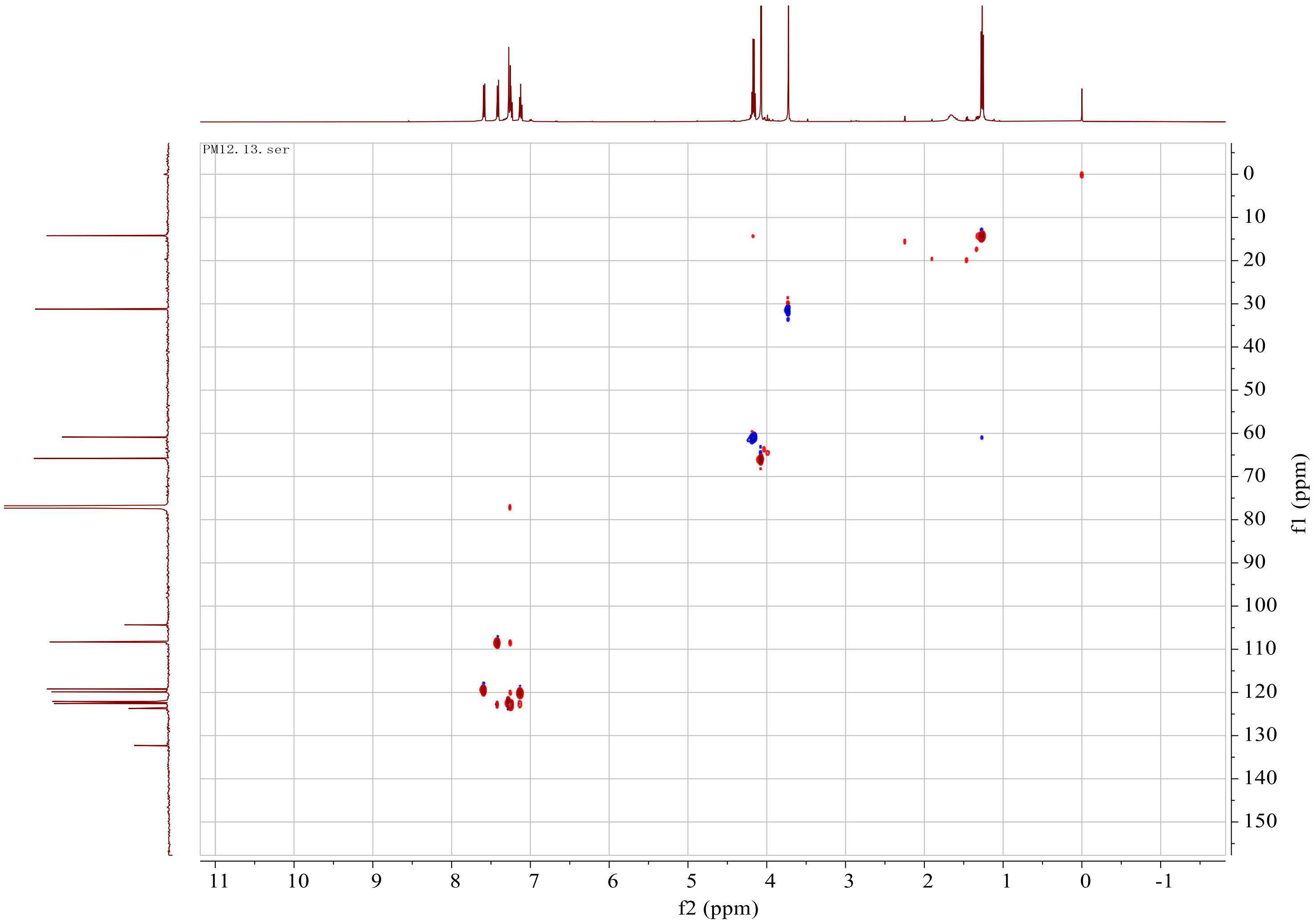


**Figure S9.** HSQC spectrum of ethyl 2-(1-methoxy-1H-indol-3-yl) acetate (**2**) ($CDCl_3$)

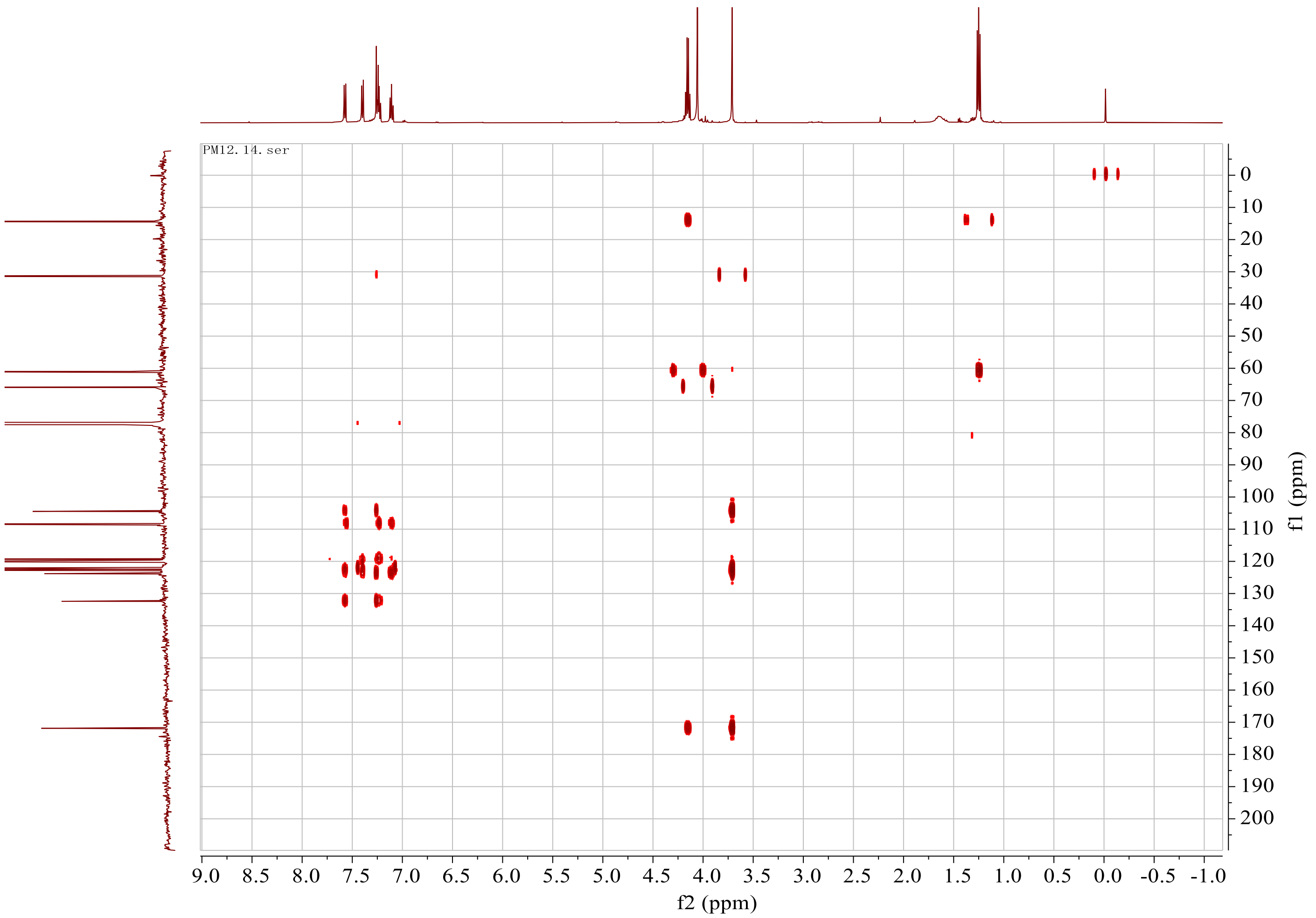


**Figure S10.** HMBC spectrum of ethyl 2-(1-methoxy-1H-indol-3-yl) acetate (**2**) ($CDCl_3$)

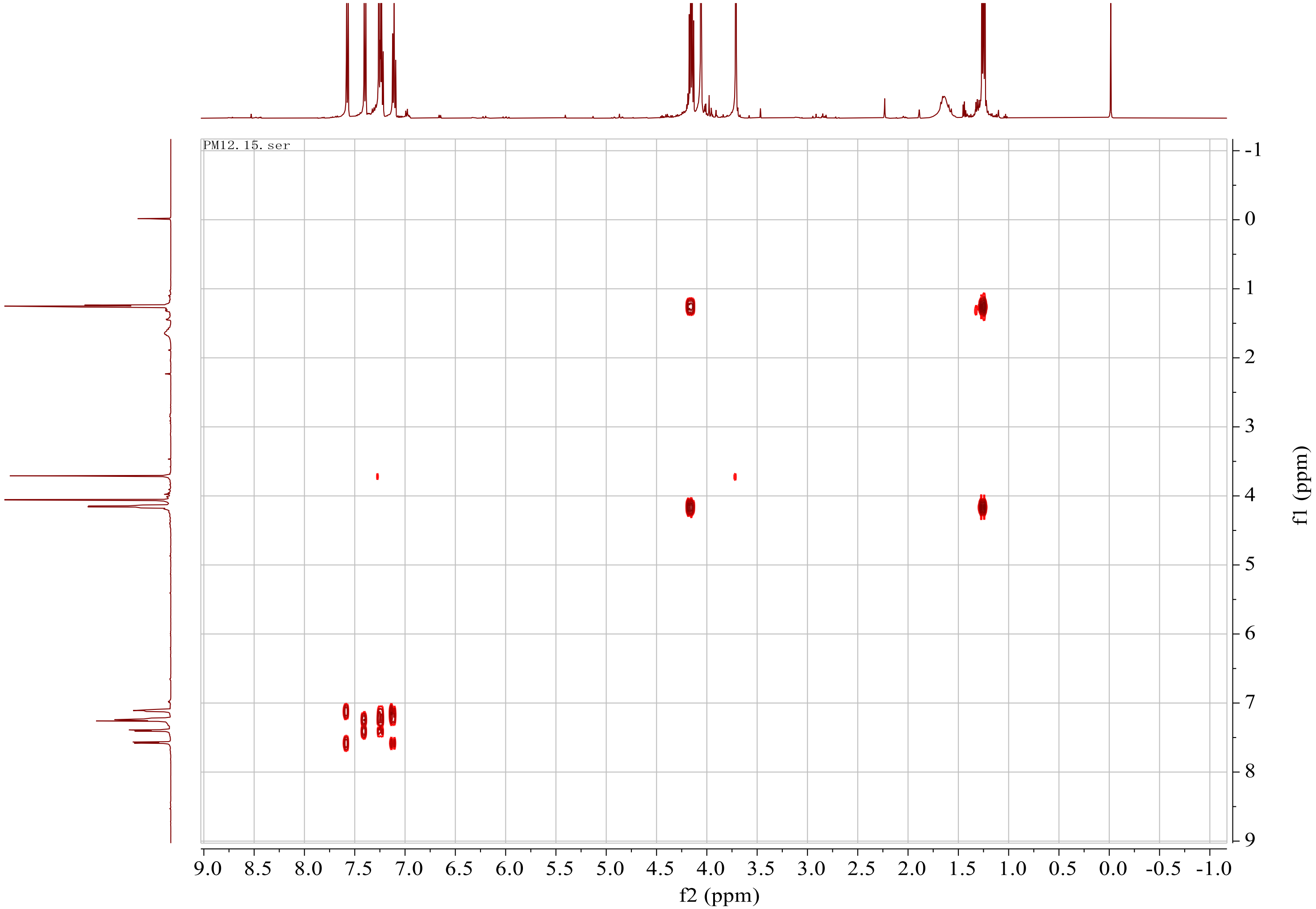


**Figure S11.** $^{1}H$-$^{1}H$ COSY spectrum of ethyl 2-(1-methoxy-1H-indol-3-yl) acetate (**2**) ($CDCl_3$)

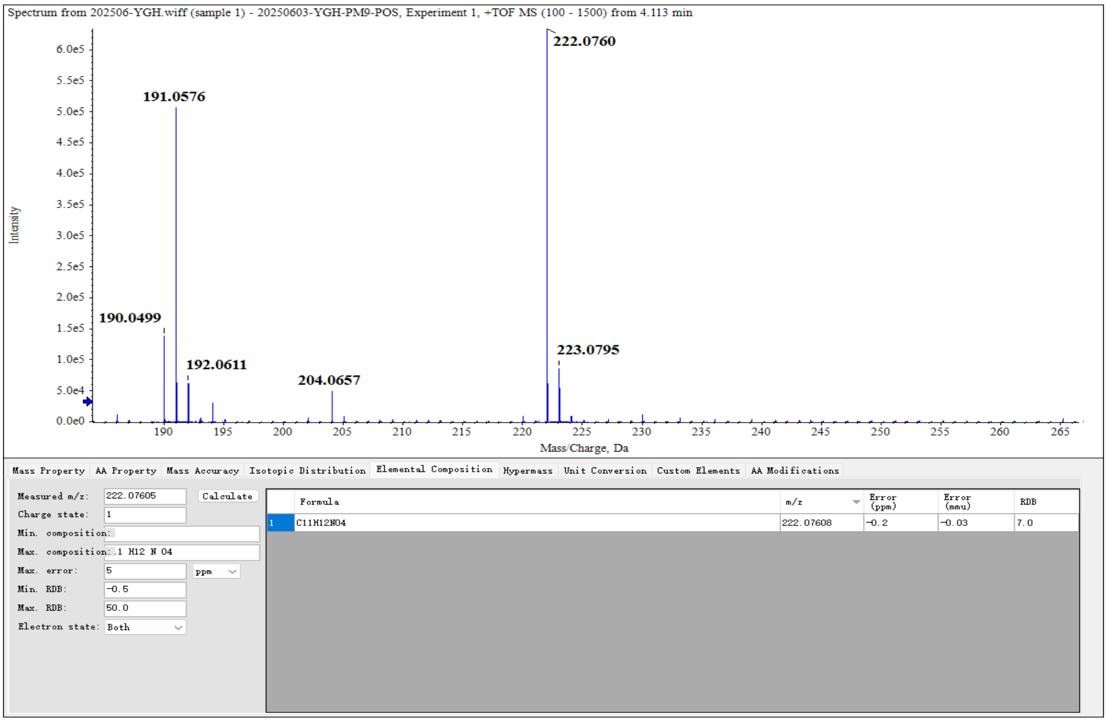


**Figure S12.** HR-ESIMS spectrum of furoindolin A (**1**)

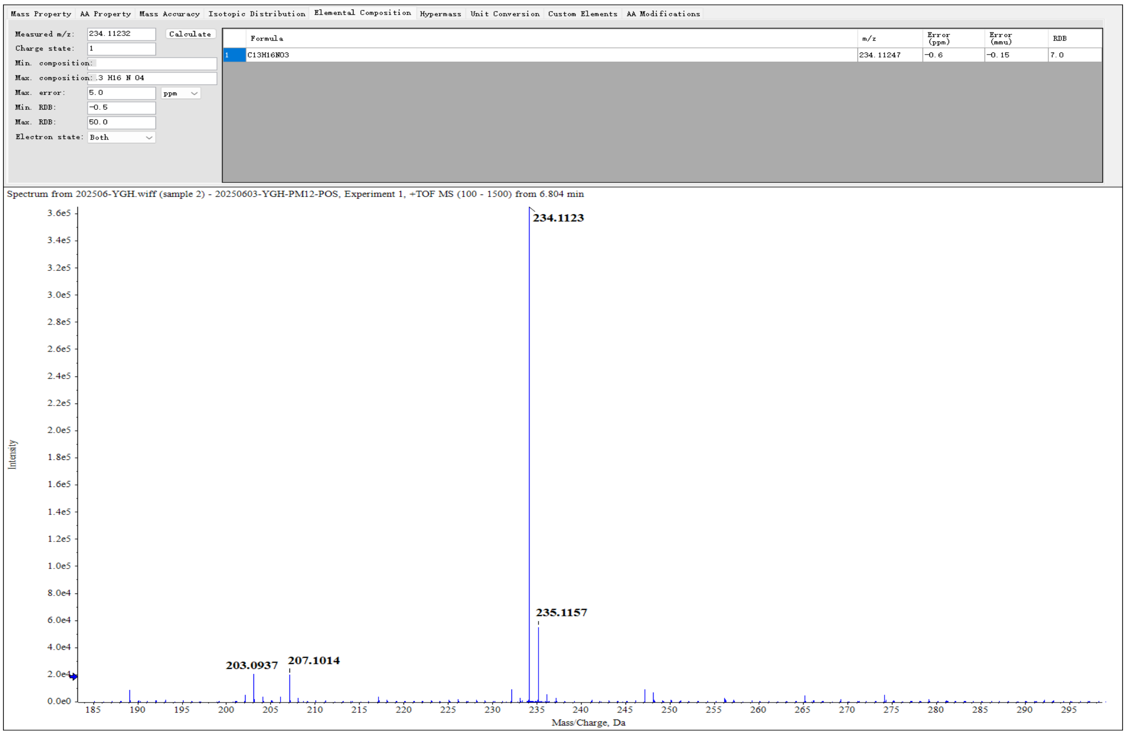


**Figure S13.** HR-ESIMS spectrum of ethyl 2-(1-methoxy-1*H*-indol-3-yl) acetate (**2**)

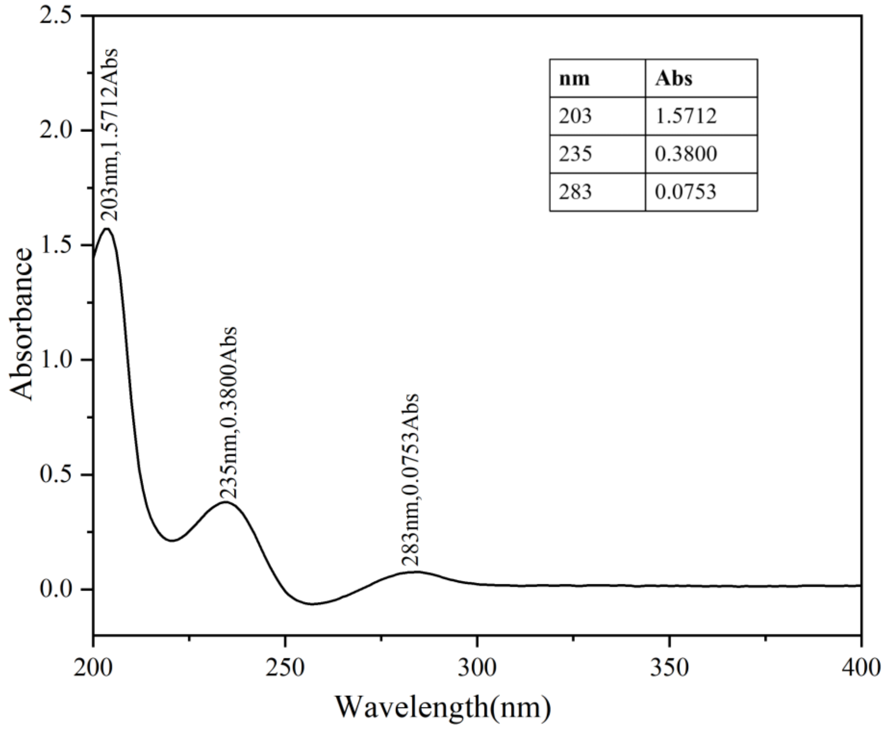


**Figure S14.** UV spectrum of furoindolin A (**1**)

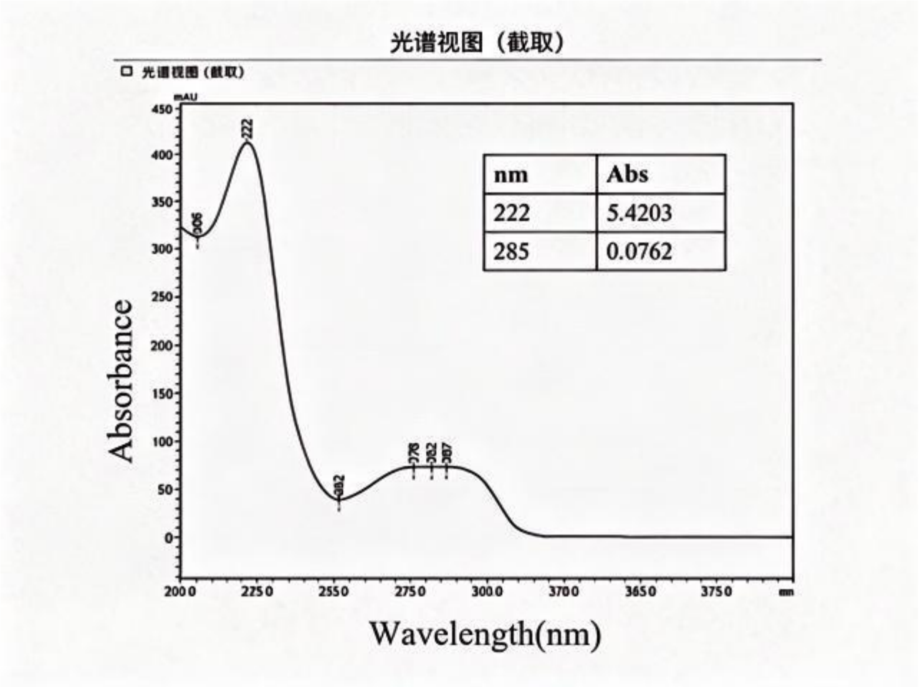


**Figure S15.** UV spectrum of ethyl 2-(1-methoxy-1*H*-indol-3-yl) acetate (**2**)

**Table S1**. Energies of **1** at MMFF94 force field.

| Configuration | Conformer | Energy (kcal/mol) | Population (%) |
|---|---|---|---|
| (3a*S*, 8a*R*)-**1** | 1 | 184.67 | 87.0 |
| (3a*S*, 8a*R*)-**1** | 2 | 189.66 | 11.6 |
| (3a*S*, 8a*R*)-**1** | 3 | 194.98 | 1.4 |
| (3a*S*, 8a*R*)-**1** | 4 | 208.98 | 0.0 |
| (3a*R*, 8a*S*)-**1** | 1 | 184.67 | 87.0 |
| (3a*R*, 8a*S*)-**1** | 2 | 189.66 | 11.6 |
| (3a*R*, 8a*S*)-**1** | 3 | 194.98 | 1.4 |
| (3a*R*, 8a*S*)-**1** | 4 | 208.98 | 0.0 |

**Table S2**. Energies of **1** at B3LYP/6–31+g(d) level in methanol.

| Configuration | Conformer | E (Hartree) | E (kcal/mol) | Population (%) |
|---|---|---|---|---|
| (3a*S*, 8a*R*)-**1** | 1 | –781.3993919 | –490335.932411169 | 85.77 |
| (3a*S*, 8a*R*)-**1** | 2 | –781.39686 | –490334.3436186 | 5.86 |
| (3a*S*, 8a*R*)-**1** | 3 | –781.3970356 | –490334.453809356 | 7.05 |
| (3a*S*, 8a*R*)-**1** | 4 | –781.3954563 | –490333.462782813 | 1.32 |
| (3a*R*, 8a*S*)-**1** | 1 | –781.3993918 | –490335.932348418 | 85.76 |
| (3a*R*, 8a*S*)-**1** | 2 | –781.39686 | –490334.3436186 | 5.86 |
| (3a*R*, 8a*S*)-**1** | 3 | –781.3970357 | –490334.453872107 | 7.06 |
| (3a*R*, 8a*S*)-**1** | 4 | –781.3954568 | –490333.463096568 | 1.32 |

(3a*S*, 8a*R*)-**1**

| | |
|---|---|
| 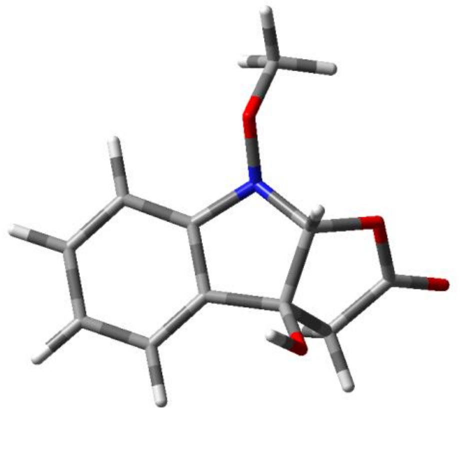 | 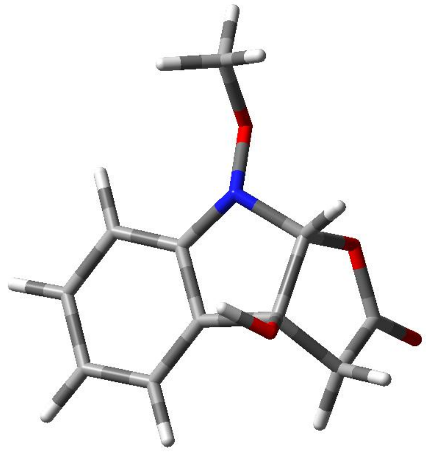 |
| Conf.1 (85.77%) | Conf.2 (5.86%) |
| 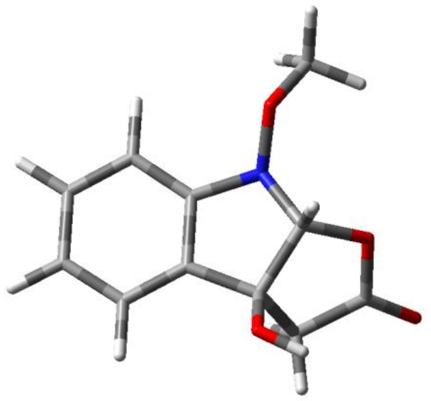 | 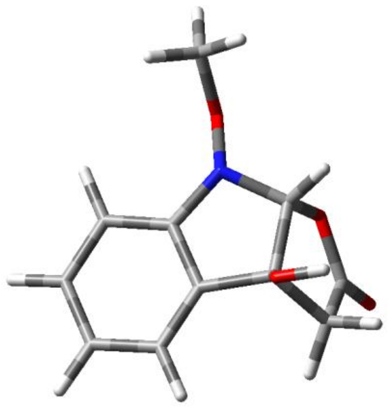 |
| Conf.3 (7.05%) | Conf.4 (1.32%) |

(3a*R*, 8a*S*)-**1**

| | |
|---|---|
| 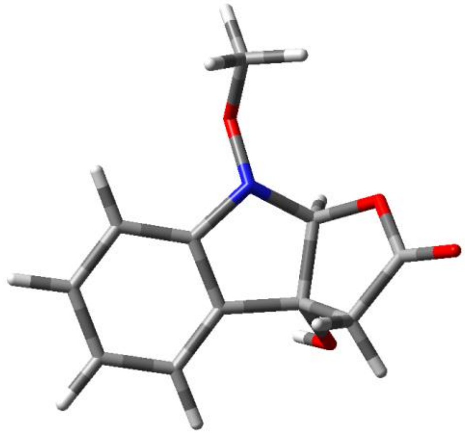 | 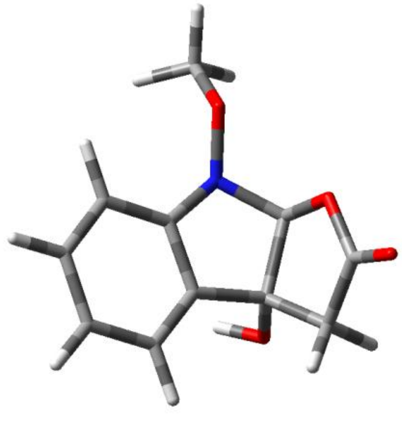 |
| Conf.1 (85.76%) | Conf.2 (5.86%) |

| | |
|---|---|
| 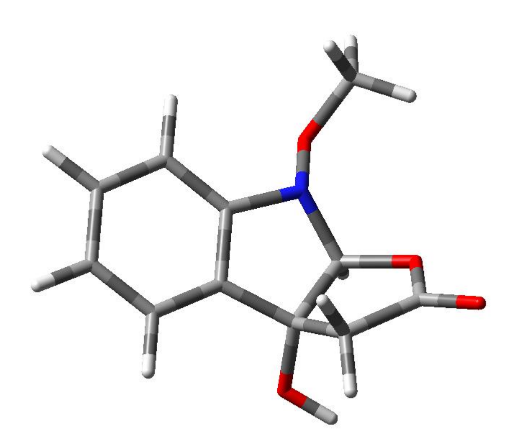 | 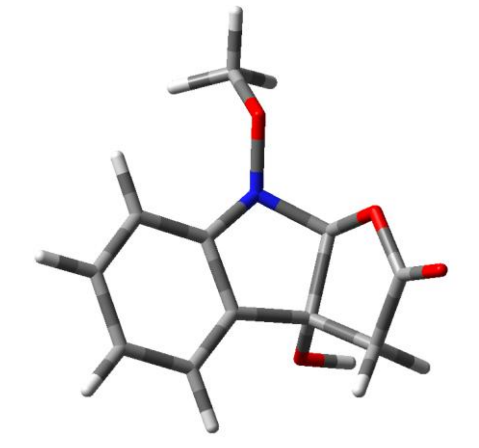 |
| Conf.3 (7.06%) | Conf.4 (1.32%) |

**Figure S16.** The optimized conformers and equilibrium populations of **1**.

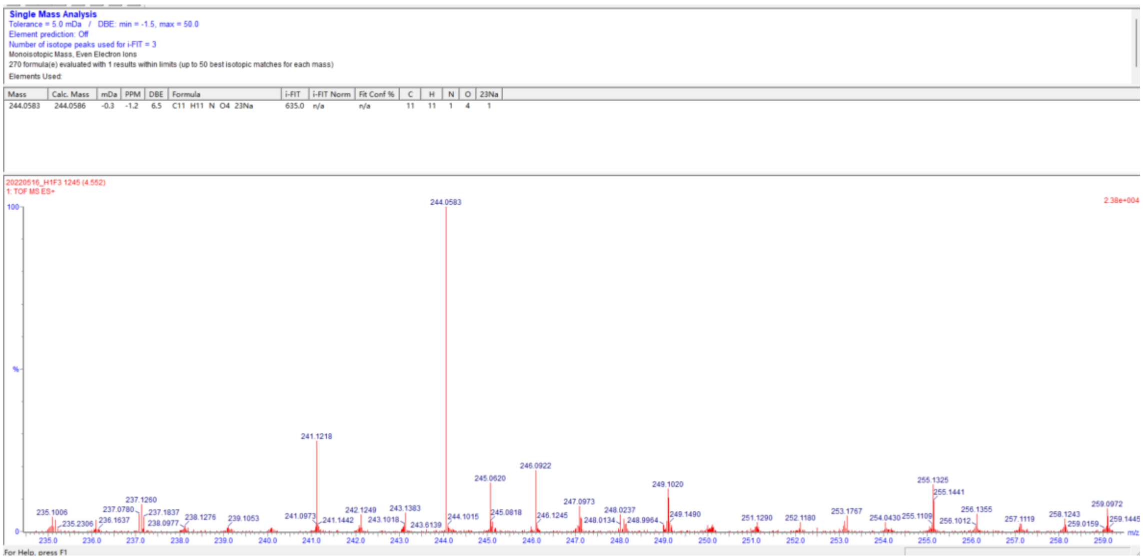


**Figure S17**. The HR-ESI-MS ion peak of furoindolin A (**1**) from LC-MS data of the crude extracts of the strain *P. microspora* GXIMD 02530.

**Physicochemical data of known compounds 3–16.**

1-Methoxyindole-3-acetate (**3**): white powder; $^{1}$H NMR (500 MHz, $CDCl_3$): $\delta_H$ 7.57 (1H, dt, $J$ = 8.0, 0.9 Hz, H-4), 7.41 (1H, dt, $J$ = 8.1, 0.9 Hz, H-7), 7.24 (1H, ddd, $J$ = 8.0, 7.1, 1.0 Hz, H-6), 7.12 (1H, ddd, $J$ = 8.0, 7.1, 1.0 Hz, H-5), 4.06 (3H, s, H-11), 3.73 (2H, s, H-3), 3.70 (3H, s, H-1'). $^{13}$C NMR (125 MHz, $CDCl_3$): $\delta_C$ 172.3 (C, C-2), 132.4 (C, C-7a), 123.8 (C, C-3b), 122.7 (CH, C-8a), 122.2 (CH, C-6), 120.0 (CH, C-5), 119.2 (CH, C-4), 108.5 (CH, C-7), 104.3 (C, C-3a), 66.0 (-$OCH_3$, C-1'), 52.1 (-$OCH_3$, C-11), 31.1 ($CH_2$, C-3).

Methyl indole-3-acetate (**4**): white powder; $^1$H NMR (500 MHz, $CDCl_3$): $\delta_H$ 8.09 (1H, s, H-11), 7.62 (1H, d, $J$ = 7.9 Hz, H-4), 7.37 (1H, d, $J$ = 8.1 Hz, H-7), 7.20 (2H, m, H-6), 7.15 (1H, m, H-5), 3.79 (2H, s, H-3), 3.71 (3H, s, H-1'). $^{13}$C NMR (125 MHz, $CDCl_3$): $\delta_C$ 172.7 (C, C-2), 136.2 (C, C-7a), 127.4 (C, C-3b), 123.2 (CH, C-8a), 122.4 (CH, C-6), 119.9 (CH, C-5), 119.0 (CH, C-4), 111.3 (CH, C-7), 108.7 (C, C-3a), 52.1 (-$OCH_3$, C-1'), 31.3 ($CH_2$, C-3).

1*H*-indole-3-carboxaldehyde (**5**): pale yellow powder; $^1$H NMR (500 MHz, DMSO-$d_6$): $\delta_H$ 12.13 (1H, s, H-1), 9.94 (1H, s, H-10), 8.28 (1H, s, H-2), 8.10 (1H, d, $J$ = 7.6 Hz, H-4), 7.51 (1H, d, $J$ = 7.6 Hz, H-7), 7.24 (2H, m, H-5, 6). $^{13}$C NMR (125 MHz, DMSO-$d_6$): $\delta_C$ 185.0 (-CHO, C-10), 138.4 (CH, C-2), 137.1 (C, C-8), 124.1 (C, C-9), 123.5 (CH, C-4), 122.1 (CH, C-5), 120.8 (CH, C-6), 118.2 (C, C-3), 112.4 (CH, C-7).

5,7-Dimethoxy-4,6-dime-thylphthalide (**6**): colorless oil; $^1$H NMR (500 MHz, Chloroform-*d*): $\delta_H$ 5.11 (2H, s, H-3), 4.01 (3H, s, H-12), 3.75 (3H, s, H-10), 2.21 (3H, s, H-13), 2.16 (3H, s, H-11). $^{13}$C NMR (125 MHz, $CDCl_3$): $\delta_C$ 169.1 (C, C-1), 163.0 (C, C-6), 156.7 (C, C-8), 146.2 (C, C-4), 125.4 (C, C-7), 120.0 (C, C-9), 112.6 (C, C-5), 68.4 ($CH_2$, C-3), 62.3 ($CH_3$, C-10), 60.3 ($CH_3$, C-12), 11.4 ($CH_3$, C-13), 9.4 ($CH_3$, C-11).

7-hydroxy-5-methoxy-4,6-dimethylisobenzofuran-1(3*H*)-one (**7**): red-brown solid; $^1$H NMR (500 MHz, DMSO-$d_6$): $\delta_H$ 12.13 (1H, s, H-1), 9.94 (1H, s, H-10), 8.28 (1H, s, H-2), 8.10 (1H, d, $J$ = 7.6 Hz, H-4), 7.51 (1H, d, $J$ = 7.6 Hz, H-7), 7.24 (2H, m, H-5, 6). $^{13}$C NMR (125 MHz, DMSO-$d_6$): $\delta_C$ 185.0 (-CHO, C-10), 138.4 (CH, C-2), 137.1 (C, C-8), 124.1 (C, C-9), 123.5 (CH, C-4), 122.1 (CH, C-5), 120.8 (CH, C-6), 118.2 (C, C-3), 112.4 (CH, C-7).

Pestalotiophthalide B (**8**): white solid; $^1$H NMR (500 MHz, Chloroform-*d*): $\delta_H$ 5.28 (2H, s, H-3), 5.15 (2H, s, H-1'), 4.07 (3H, s, H-6'), 3.83 (3H, s, H-4'), 2.24 (3H, s, H-5'), 2.09 (3H, s, H-3'). $^{13}$C NMR (125 MHz, $CDCl_3$): $\delta_C$ 170.7 (C, C-2'), 168.5 (C, C-1), 163.6 (C, C-5), 159.2 (C, C-7), 147.2 (C, C-3a), 125.9 (C, C-6), 118.7 (C, C-4), 113.2 (C, C-7a), 68.5 ($CH_2$, C-3), 62.5 ($CH_3$, C-6'), 61.8 ($CH_3$, C-4'), 58.5 ($CH_2$, C-1'),

20.9 ($CH_3$, C-3'), 9.6 ($CH_3$, C-5').

Gamahorin (**9**): colorless syrup; ECD (0.2 mg/mL , MeOH) $\lambda_{max}$ ($\Delta\varepsilon$) 246 (+7.99), 213 (+1.67), 205 (-6.39) nm; $^1$H NMR (500 MHz, $CDCl_3$): $\delta_H$ 11.39 (1H, s, 3-OH), 7.31 (1H, d, $J$ = 7.6 Hz, H-5), 6.67 (1H, d, $J$ = 7.6 Hz, H-6), 4.47 (1H, m, H-8), 2.84 (1H, m, H-7), 2.24 (3H, s, $H_3$-13), 1.46 (3H, d, $J$ = 6.4 Hz, $H_3$-12), 1.33 (3H, d, $J$ = 7.0 Hz, $H_3$-11). $^{13}$C NMR (125 MHz, $CDCl_3$): $\delta_C$ 169.8 (C, C-2), 160.6 (C, C-3), 141.3 (C, C-10), 137.2 (CH, C-5), 125.3 (C, C-4), 115.9 (CH, C-6), 107.0 (C, C-9), 81.2 (CH, C-8), 37.3 (CH, C-7), 19.8 ($CH_3$, C-12), 17.3 ($CH_3$, C-11), 15.5 ($CH_3$, C-13).

Phomopsilactone (**10**): yellow solid; ECD (0.2 mg/mL , MeOH) $\lambda_{max}$ ($\Delta\varepsilon$) 257 (+108.99), 222 (-28.67) nm; $^1$H NMR (500 MHz, $CDCl_3$): $\delta_H$ 12.79 (1H, s, 3-OH), 11.87 (1H, s, 5-OH), 10.11 (1H, s, H-14), 4.89 (1H, d, $J$ = 2.4 Hz, H-12a), 4.68 (1H, d, $J$ = 2.3 Hz, H-12b), 4.30 (1H, q, $J$ = 7.2 Hz, H-7), 2.08 (3H, s, H-13), 1.56 (3H, d, $J$ = 7.3 Hz, H-11). $^{13}$C NMR (125 MHz, $CDCl_3$): $\delta_C$ 191.5 (-CHO, C-14), 167.6 (C, C-5), 166.1 (C, C-2), 166.1 (C, C-3), 155.0 (C, C-8), 148.8 (C, C-10), 112.2 (C, C-4), 108.9 (C, C-6), 99.7 (C, C-9), 97.6 ($CH_2$, C-12), 32.6 (CH, C-7), 25.2 ($CH_3$, C-11), 7.0 ($CH_3$, C-13).

Pestalopyrone (**11**): white powder; $^1$H NMR (500 MHz, Chloroform-*d*): $\delta_H$ 6.66 (1H, m, H-8), 5.88 (1H, d, $J$ = 2.2 Hz, H-5), 5.43 (1H, d, $J$ = 2.2 Hz, H-3), 3.79 (3H, s, H-11), 1.81 (6H, m, H-9, 10). $^{13}$C NMR (125 MHz, $CDCl_3$): $\delta_C$ 171.6 (C, C-4), 164.4 (C, C-2), 161.4 (C, C-6), 130.3 (CH, C-8), 126.7 (C, C-7), 97.3 (CH, C-5), 88.1 (CH, C-3), 56.0 (-$OCH_3$, C-11), 14.3 ($CH_3$, C-9), 12.1 ($CH_3$, C-10).

Cladoacetal A (**12**): white solid; ECD (0.2 mg/mL , MeOH) $\lambda_{max}$ ($\Delta\varepsilon$) 266 (+23.61), 224 (+2.10), 211 (+3.29), 203 (−3.45) nm; $^1$H NMR (500 MHz, $CDCl_3$): $\delta_H$ 6.99, dd (1H, dd, $J$ = 8.3, 7.5 Hz, H-8), 6.78 (1H, s, H-1), 6.77 (1H, m, H-7), 6.47 (1H, m, H-9), 5.82 (1H, dd, $J$ = 11.9, 5.0 Hz, H-5), 4.65, (1H, dd, $J$ = 4.8, 4.5 Hz, H-4), 4.44 (1H, m, H-3), 1.35 (3H, d, $J$ = 6.4 Hz, H-11). $^{13}$C NMR (125 MHz, $CDCl_3$): $\delta_C$ 153.5 (C, C-10), 136.2 (C, C-6a), 132.7 (CH, C-6), 129.6 (CH, C-5), 129.5 (CH, C-8), 125.6 (C, C-10a), 125.5 (CH, C-7), 116.1 (CH, C-9), 98.9 (CH, C-1), 84.9 (CH, C-3), 78.1 (CH, C-4), 13.7 ($CH_3$, C-11).

Clavisinol E (**13**): brown amorphous solid; ECD (0.2 mg/mL , MeOH) $\lambda_{max}$ ($\Delta\varepsilon$) 234 (+2.10), 203 (-1.85) nm;$^{1}$H NMR (500 MHz, $CDCl_3$): $\delta_H$ 11.86 (1H, s, 3-OH), 10.32 (1H, s, H-1), 7.43 (1H, t, *J* = 8.0 Hz, H-5), 6.95 (1H, d, *J* = 15.6 Hz, H-8), 6.92 (1H, d, *J* = 7.5 Hz, H-6), 6.86 (1H, d, *J* = 8.4 Hz, H-4), 6.14 (1H, dt, *J* = 15.1, 7.3 Hz, H-9), 3.99 (1H, m, H-11), 2.44 (2H, m, H-10), 1.28 (3H, d, *J* = 6.2 Hz, H-12). $^{13}$C NMR (125 MHz, $CDCl_3$): $\delta_C$ 195.6 (-CHO, C-1), 162.9 (C, C-3), 143.1 (C, C-7), 137.3 (CH, C-5), 134.6 (CH, C-9), 127.5 (CH, C-8), 118.9 (CH, C-6), 117.4 (C, C-2), 116.9 (CH, C-4), 67.5 (CH, C-11), 43.1 ($CH_2$, C-10), 23.4 ($CH_3$, C-12).

Methyl-(2-formyl-3-hydroxyphenyl) propanoate (**14**): white solid; ECD (0.2 mg/mL , MeOH) $\lambda_{max}$ ($\Delta\varepsilon$) 217 (-2.77), 214 (+0.50), nm; $^1$H NMR (500 MHz, $CDCl_3$): $\delta_H$ 11.98 (1H, s,3-OH), 10.35 (1H, s, H-1), 7.41 (1H, m, H-5), 6.85 (1H, d, *J* = 8.4 Hz, H-6), 6.76 (1H, d, *J* = 7.5 Hz, H-4), 3.68 (3H, s, H-12), 3.27 (2H, t, *J* = 7.8 Hz, H-8), 2.67 (2H, t, *J* = 7.8 Hz, H-9). $^{13}$C NMR (125 MHz, $CDCl_3$): $\delta_C$ 194.9 (CH, C-1), 172.5 (C, C-10), 163.6 (C, C-3), 144.8 (C, C-7), 137.7 (CH, C-5), 120.9 (CH, C-6), 117.9 (C, C-2), 116.9 (CH, C-4), 52.0 ($CH_3$, C-12), 36.4 ($CH_2$, C-9), 26.4 ($CH_2$, C-8).

3-(2-Formyl-3-hydroxyphenyl)-propionic acid (**15**): white solid; $^1$H NMR (500 MHz, CDCl3): $\delta_H$ 11.97 (1H, s, H-10), 10.34 (1H, s, H-1), 7.42 (1H, dd, J = 8.4, 7.4 Hz, H-5), 6.85 (1H, d, J = 8.4 Hz, H-4), 6.77 (1H, d, J = 7.5 Hz, H-6), 3.27 (2H, t, J = 7.7 Hz, H-8), 2.73 (2H, t, J = 7.7 Hz, H-9). $^{13}$C NMR (125 MHz, CDCl3): $\delta_C$ 194.9 (-CHO, C-1), 178.2 (C, C-10), 163.5 (C, C-3), 144.4 (C, C-7), 137.7 (CH, C-5), 120.8 (CH, C-6), 117.8 (C, C-2), 117.0 (CH, C-4), 36.1 (CH2, C-9), 25.9 (CH2, C-8).

2-*P*-acetoxyphenylethanol (**16):** white solid; $^1$H NMR (500 MHz, Chloroform-*d*): $\delta_H$ 7.05 (d, *J* = 8.5 Hz, H-2', H-6'), 6.80 (d, *J* = 8.8 Hz, H-3', H-5'), 4.25 (t, *J* = 7.1 Hz, H-1), 2.86 (t, *J* = 7.1 Hz, H-2), 2.05 (s, H-4). $^{13}$C NMR (126 MHz, $CDCl_3$): $\delta_C$ 172.3(C, C-3), 154.7(C, C-5'), 130.1(CH, C-2', C-6'), 115.5(CH, C-3', C-5'), 65.7($CH_2$, C-1), 34.1($CH_2$, C-2), 21.0($CH_3$, C-4).

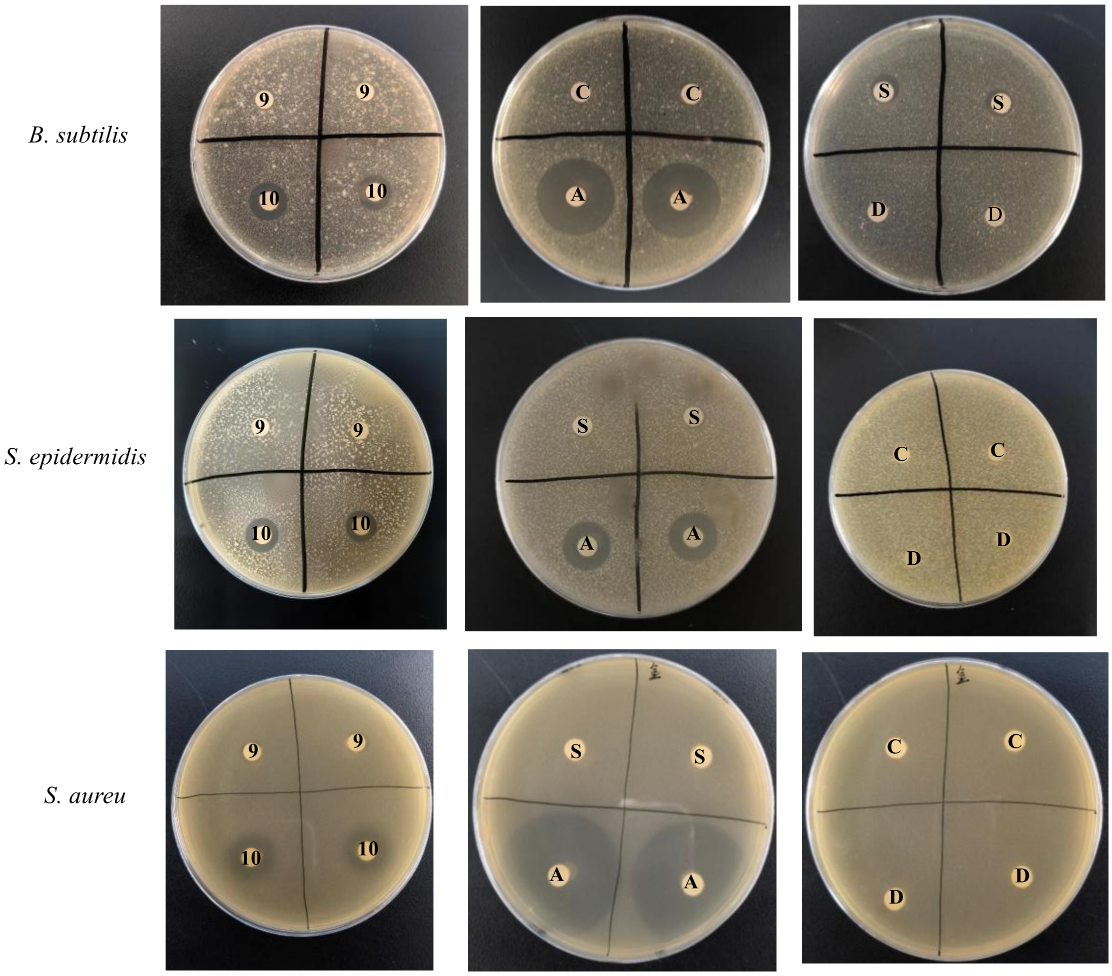


**Figure S18.** Inhibition zone diameter range (mm) of compounds **9-10** and positive controls against *B. subtilis*, *S. epidermidis*, and *S. aureus* after 24 h of incubation at 37 °C. [A (ampicillin), C (chloramphenicol), and S (streptomycin) served as positive controls, while D represents the DMSO solvent control]

**The ITS sequence of *Pestalotiopsis microspora* GXIMD 02530**

TGTGACTTACCATTGTTGCCTCGGCAGAAGCTGCTTGGCGCGCCTTACCTT
GGAACGGCCTACCCTGTAGCGCCTTACCCTGGAACGGCTTACCCTGCAACG
GCTGCCGGTGGACTACCAAACTCTTGTTATTTATGGTTATCTGAGCGTCTT
ATTTTAATAAGTCAAAACTTTCAACAACGGATCTCTTGGTTCTGGCATCGAT
GAAGAACGCAGCGAAATGCGATAAGTAATGTGAATTGCAGAATTCAGTGA
ATCATCGAATCTTTGAACGCACATTGCGCCCATTAGTATTCTAGTGGGCATG
CCTGTTCGAGCGTCATTTCAACCCTTAAGCCTAGCTTAGTGTTGGGAGCCTA
CTGCTTTTGCTAGCTGTAGCTCCTGAAATACAACGGCGGATCTGCGATATCC
TCTGAGCGTAGTAATTTTTATCTCGCTTTTGACTGGAGTTGCAGCGTCTTTA
GCCGCTAAACCCCCCAATTTTTAATGG
